# Molecular cloning of the tomato *Hairless* gene implicates actin dynamics in trichome-mediated defense and mechanical properties of stem tissue

**DOI:** 10.1093/jxb/erw292

**Published:** 2016-07-31

**Authors:** Jin-Ho Kang, Marcelo L. Campos, Starla Zemelis-Durfee, Jameel M. Al-Haddad, A. Daniel Jones, Frank W. Telewski, Federica Brandizzi, Gregg A. Howe

**Affiliations:** ^1^Department of Energy-Plant Research Laboratory, Michigan State University, East Lansing, MI 48824, USA; ^2^Graduate School of International Agricultural Technology and Crop Biotechnology Institute/GreenBio Science and Technology, Seoul National University, Pyeongchang 25354, Republic of Korea; ^3^Department of Plant Biology, Michigan State University, East Lansing, MI 48824, USA; ^4^Department of Chemistry, Michigan State University, East Lansing, MI 48824, USA; ^5^Department of Biochemistry and Molecular Biology, Michigan State University, East Lansing, MI 48824, USA

**Keywords:** Actin-cytoskeleton network, cell wall, mechanical properties, plant-insect interaction, specialized metabolism, terpenoid, tomato (*Solanum lycopersicum*), trichome.

## Abstract

Trichomes are epidermal structures that provide a first line of defense against arthropod herbivores. The recessive *hairless* (*hl*) mutation in tomato (*Solanum lycopersicum* L.) causes severe distortion of trichomes on all aerial tissues, impairs the accumulation of sesquiterpene and polyphenolic compounds in glandular trichomes, and compromises resistance to the specialist herbivore *Manduca sexta*. Here, we demonstrate that the tomato *Hl* gene encodes a subunit (SRA1) of the highly conserved WAVE regulatory complex that controls nucleation of actin filaments in a wide range of eukaryotic cells. The tomato *SRA1* gene spans a 42-kb region containing both *Solyc11g013280* and *Solyc11g013290*. The *hl* mutation corresponds to a complex 3-kb deletion that removes the last exon of the gene. Expression of a wild-type *SRA1* cDNA in the *hl* mutant background restored normal trichome development, accumulation of glandular trichome-derived metabolites, and resistance to insect herbivory. These findings establish a role for *SRA1* in the development of tomato trichomes and also implicate the actin-cytoskeleton network in cytosolic control of specialized metabolism for plant defense. We also show that the brittleness of *hl* mutant stems is associated with altered mechanical and cell morphological properties of stem tissue, and demonstrate that this defect is directly linked to the mutation in *SRA1*.

## Highlight

This study implicates actin dynamics in glandular trichome development, cytosolic control of specialized metabolism, and mechanical properties of plant tissues.

## Introduction

Trichomes are epidermal outgrowths that exhibit remarkable morphological variation between plant species. In addition to being either unicellular or multicellular, trichomes are often distinguished as being nonglandular (simple hairs) or glandular, with the latter involved in the production and secretion of a wide spectrum of specialized metabolites. Trichomes have been implicated in several physiological functions related to biotic and abiotic stress resilience. For example, trichomes protect plants against a variety of arthropod herbivores that depend on plants as a source of nutrition, prevent water loss and may also provide protection from UV-B radiation (Karabourniotis *et al.*, 1992; Werker, 2000; Kennedy, 2003).

Molecular genetic studies performed in Arabidopsis have provided detailed insight into the regulatory pathways that control the development of simple, unicellular trichomes. These studies have identified multi-protein transcriptional complexes that control the differentiation of trichomes from epidermal pavement cells (Hulskamp *et al.*, 1994; Marks, 1997; Ramsay and Glover, 2005; Yang and Ye, 2013). Characterization of trichome distortion mutants has further revealed a role for the actin-cytoskeleton network in trichome branching, cell expansion, and other aspects of cell morphogenesis (Mathur and Chua, 2000; Mathur *et al.*, 2003; Szymanski, 2005). Increasing interest in multicellular glandular trichomes as ‘biofactories’ for the production of specialized metabolites has stimulated efforts to understand developmental and metabolic processes that define these more complex epidermal structures and their role in plant interactions with the environment (Schilmiller *et al.*, 2008; Bergau *et al.*, 2015). The extent to which mechanisms underlying the developmental control of trichomes in Arabidopsis applies to multicellular glandular trichomes, however, is largely unknown.

Cultivated tomato (*Solanum lycopersicum* L.) and its wild relatives have several morphologically distinct trichome types, including both non-glandular and glandular structures (Luckwill, 1943). Among the best studied of these are the type I and VI glandular trichomes. Type I trichomes are characterized by a multicellular base, a long (~2mm) multicellular stalk, and a small glandular tip that secretes acyl sugars (Goffreda *et al.*, 1990; Schilmiller *et al.*, 2012). The type VI trichome has a short (~0.1mm) stalk and a four-celled glandular head in which high concentrations of terpenoids and flavonoids are produced (Kennedy, 2003; Kang *et al.*, 2010*a*; Bergau *et al.*, 2015). Recent advances in ‘omics’ technologies have helped to elucidate the biochemical pathways that underlie the production of these compounds (Sallaud *et al.*, 2009; Schilmiller *et al.*, 2009, 2012; McDowell *et al.*, 2011). Complementary genetic approaches have provided evidence for a link between glandular trichome chemistry and resistance of cultivated tomato to arthropod herbivores (Kang *et al.*, 2010*a*, *b*; Tian *et al.*, 2012). In only a few cases, however, have genes that are required for proper development and function of glandular trichomes been identified. For example, the *jai-1* mutation defines a component (CORONATINE-INSENSITIVE1) of the jasmonate (JA) receptor whose activity modulates the density and metabolic output of glandular type VI trichomes (Li *et al.*, 2004*a*; Katsir *et al.*, 2008). It has also been shown that loss-of-function of the flavonoid pathway enzyme chalcone isomerase (CHI1) is associated with reduced density and metabolic output of type VI trichome glands (Rick *et al.*, 1976; Kang *et al.*, 2014). In contrast, downregulation of the methyl-D-erythritol 4-phosphate (MEP) pathway enzyme 1-deoxy-*D*-xylulose 5-phosphate synthase 2 (DXH2) increases type VI trichome density (Paetzold *et al.*, 2010). The tomato *Woolly* gene encodes a homeodomain-containing transcription factor that specifically controls the development of type I trichomes (Yang *et al.*, 2011). These collective findings indicate that the development of various trichome types in tomato involves regulatory processes that are specific to a particular structure. A better understanding of trichome development and function could be achieved through the study of genes that affect multiple trichome types in a single plant species.

**Fig. 1.**
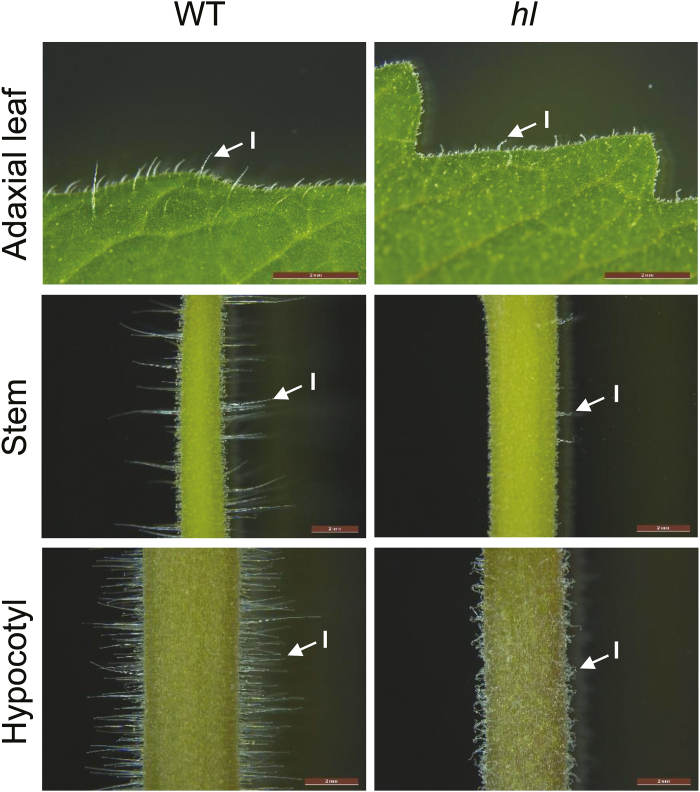
Light micrographs of trichomes on the leaf, stem, and hypocotyl of WT and *hl* plants. Photographs show the adaxial leaf surface (upper row), stem (middle row), and hypocotyl (lower row) of each genotype. Arrows indicate representative type I trichomes. Scale bar, 2mm. Three-week-old plants were used for all photographs. (This figure is available in color at *JXB* online.)

The *hairless* (*hl*) mutant of tomato was identified more than a half-century ago as a spontaneous mutant that is defective in trichome production on stems and hypocotyls (Rick and Butler, 1956). Reeves (1977) noted that the ‘hairless’ phenotype of the mutant is not caused by the absence of trichomes but rather the development of trichomes that are severely bent and shortened, which imparts a granular appearance to epidermal surfaces (Fig. 1). Subsequent studies showed that *hl* plants are deficient in the accumulation of sesquiterpene and polyphenolic compounds within type VI glandular trichomes and that this metabolic phenotype is associated with decreased resistance to insect herbivory (Kang *et al.*, 2010*b*). Here, we used a map-based cloning approach to demonstrate that *Hl* encodes the highly conserved SRA1 (specifically Rac1-associated protein) subunit of the WAVE regulatory complex (WRC), which is known to control branching of actin filaments in response to various signal inputs at the cell surface. Transgenic complementation experiments showed that *SRA1* is required for normal development of all trichome types, accumulation of flavonoids and sesquiterpenoids in type VI glands, and resistance to *M. sexta*. These findings reveal a function for *SRA1* in glandular trichome development and further suggest that actin-cytoskeleton dynamics play a role in the production of cytosolic metabolites that confer resistance to insect herbivores. We also demonstrate that the *hl* mutation is responsible for the brittleness of mutant stems, thus establishing a role for SRA1 in mechanical properties of tomato tissues.

## Materials and methods

### Plant materials and growth conditions

Tomato (*Solanum lycopersicum*) cv Alisa Craig (LA2838A) was used as the wild type (WT) for all experiments. Seeds for WT and *hairless* (*hl*, accession number LA3556) were obtained from CM Rick Tomato Genetics Resource Center (University of California, Davis, USA). Seedlings were grown in Jiffy peat pots (Hummert International, Earth City, MO, USA) in a growth chamber maintained under 17h of light (265 mE m^−2^ s^−1^) at 27 °C and 7h of dark at 18 °C and 60% humidity. Three- to four-week-old plants were sampled for morphological and secondary metabolite analysis.

### Genetic mapping of *Hl*

Fine mapping of *Hl* was performed with an F_2_ population derived from a cross between *hl* mutant (LA3556) and *S. pennellii* (LA0716), and was facilitated by the tomato genome sequence (Tomato Genome Consortium, 2012). A population of 376 F_2_ plants was scored for the distorted trichome phenotype and subsequently genotyped with PCR-based COS (conserved ortholog set) markers (Tomato-EXPEN 2000 map; https://solgenomics.net). In some cases, new PCR-based markers were developed by comparing orthologous *S. pennellii* and *S. lycopersicum* EST sequences obtained in the Solanum Trichome Project (http://www.trichome.msu.edu). Primer sequences used for mapping are listed in Supplementary Table S1 at *JXB* online. Genomic DNA extraction and PCR conditions were as previously described (Kang *et al.*, 2010*a*).

### PCR analysis

RNA extracted from leaves (Plant RNeasy Kit, Qiagen) was used for cDNA synthesis (Thermoscript RT-PCR system, Invitrogen) according to the manufacturer’s instructions. All primer sets used for reverse transcription (RT)-PCR, including amplification of *SRA1* cDNAs, are listed in Supplementary Table S2. PCR was performed with a DNA Engine Dyad Thermal Cycler (Bio-Rad). RT-PCR reactions (25 µl) contained 15.25 µl H_2_O, 2 µl cDNA, 0.75 µl 10 µM solution of each primer, 0.75 µl 10mM dNTP mix, 5 µl 5× KAPA buffer, and 0.5 µl KAPA DNA polymerase (KAPAHiFi HotStart, Kapa Biosystems). The amplification protocol included an initial 5-min denaturation step at 95 °C, followed by 30 cycles in which the template was denatured for 20s at 98 °C, annealed for 15s at 65 °C, and extended for 3min at 72 °C, followed by a final incubation for 5min at 72 °C. Amplified DNA products were separated on a 1% agarose gel. Amplification of transcripts for eukaryotic Initiation Factor 4A (*elF4A*) was included as a loading control. 3ʹRACE (rapid amplification of cDNA ends) was used to determine the polyadenylation sites of *SlSRA1* transcripts from WT and *hl* plants.

Genomic DNA fragments corresponding to specific regions of *SlSRA1* in WT and *hl* plants were PCR-amplified using the primer sets (gPIR1–gPIR15) listed in Supplementary Table S2. PCR reactions (20 µl) contained 6 µl H_2_O, 2 µl genomic DNA template, 1 µl of a 10 µM solution of each primer and 10 µl of Taq polymerase 2× MeanGreen Mastermix (Syzygy Biotech). Amplicons were produced by an initial 5-min denaturation step at 94 °C, followed by 30 cycles in which the template was denatured for 45s at 94 °C, annealed for 30s at 52 °C, and extended for 1min at 72 °C, followed by a final incubation for 10min at 72 °C. Amplified products were separated on a 1% agarose gel. PCR-amplified fragments were cloned into the pGEM-T-Easy plasmid vector (Promega). Automated nucleotide sequencing was performed at the Michigan State University Genomics Technology Support Facility (http://rtsf.msu.edu/).

### Transgenic plants

A pGEM-T plasmid containing a cDNA for the entire coding region of *SlSRA1* was reamplified with the PIR-SX primer set (Supplementary Table S2). The resulting fragment was digested with *Spe*I and *Xho*I to release a 3.9-kb fragment. This product was cloned into *Spe*I and *Xho*I sites of the pBI-TS binary vector, which contains the Cauliflower Mosaic Virus (CaMV) 35S promoter and nopaline synthase (NOS) terminator (Koo *et al.*, 2006; Schilmiller *et al.*, 2007), to generate pBI-*35S:SRA1*. The resulting construct was introduced into *Agrobacterium tumefaciens* strain EHA105 and used to transform *hl* cotyledon explants as described previously (Li and Howe, 2001; Li *et al.*, 2003). The presence of the T-DNA insert in independent primary (T_0_) transformants was confirmed by PCR using the *35S:SRA1* primer set (Supplementary Table S2) to amplify a fragment spanning the CaMV promoter and *SRA1* cDNA. Regenerated T_0_ transgenic plants containing the *SRA1* transgene were potted in soil and transferred to a growth chamber for preliminary analysis of trichome development. These plants were subsequently transferred to a greenhouse for collection of seed (T_1_ generation) from individual lines. T_1_ seedlings exhibiting normal trichome development were selected from lines in which the ratio of normal:distorted trichomes was ~3:1. Plants exhibiting normal trichomes were grown in a greenhouse for collection of T_2_-generation seed. Lines in which all T_2_ progeny exhibited normal trichomes were selected as being homozygous for the transgene.

### Trichome density and morphology

A dissecting microscope (Leica MZ16, Wetzlar, Germany) equipped with KL 2500 LCD light sources (Schott, Jena, Germany) and Leica DFC 290 camera (Leica, Wetzlar, Germany) was used to document trichome morphology and density as previously described (Kang *et al.*, 2010a, *b*). These measurements were performed with WT, *hl*, and *SRA1*-complemented lines grown together in the same growth chamber.

### Metabolite analysis

Leaves from 4-week-old plants were used to prepare type VI trichome exudates and measure flavonoid and terpenoid derivatives as previously described (Kang *et al.*, 2010*b*). Briefly, a defined number of type VI glands were collected with a stretched Pasteur pipette. For flavonoid analysis, glands were dissolved in 100 µl methanol:acetic acid:water (9:1:10) containing propyl-4-hydroxybenzoate as an internal standard. For terpenoid analysis, glands were dissolved in 100 µl methyl *tertiary*-butyl ether containing tetradecane as an internal standard. Quantification of metabolites was performed using HPLC/time-of-flight MS for nonvolatile metabolites and GC-MS for volatiles terpenes (Kang *et al.*, 2010*b*).

### Insect feeding trials

Tobacco hornworm (*Manduca sexta*) eggs were obtained from Carolina Biological Supply Company (Burlington, NC, USA). Eggs were hatched at 26 °C as recommended by the supplier. Hatched larvae were transferred to leaves of 5-week-old tomato plants, unless otherwise indicated. Challenged plants were maintained in a growth chamber for the duration of the feeding trial, as described in ‘Results’. As criteria to establish the first-feeding time, larvae were required to feed continuously at the same feeding site for at least 10s.

### Analysis of stem mechanical properties

Green stems from 3-month-old plants were cut into segments that were more than 14 times longer than their diameters, as required for the bending test (Niklas, 1992; Pruyn *et al.*, 2000; Liu *et al.*, 2012) and in compliance with the testing standard ASTM D143 (1994). The four-point bending test was conducted at room temperature by loading the stem segments to failure using a United^**®**^ universal testing machine (Model SFM-20, United Calibration Corporation, USA) fitted with a 20 lb load cell (89 Newton) and at a crosshead rate of 30mm min^−1^. The span length between the two supported stem ends was 99.1mm, and the distance between either of the two crosshead loading points and the nearest stem grips was 40.8mm.

### Histology

Stems from 3-month-old plants were collected from a 10–15cm region close to the apical meristem. Sections were fixed overnight in a mixture of 0.5% glutaraldehyde and 4% paraformaldehyde in 1X phosphate-buffered saline. Samples were dehydrated through a series of graded ethanol washes, cleared with Histoclear II® (Electron Microscopy Sciences, cat# 64111-04), and embedded in paraffin wax according to established protocols (Karlgren *et al.*, 2009). Embedded samples were sectioned with a Leica Microtome and sections were kept on FisherBiotech Probe-On Plus Slides at 42 °C overnight. Sample tissue on the slide was deparaffinized in xylene and brought to 70% ethanol using a graded series. For staining of cell walls, samples were stained with Safranin O and Fast Green using established procedures (Johansen, 1940). Stained slides were sealed in xylene. Iodine staining was performed with tissue samples that were collected, embedded, and sectioned as described above. Sample tissue affixed to a slide was stained with Lugol’s iodine solution (6mM iodine, 43mM KI, and 0.2N HCl). Stained samples were viewed with an upright Zeiss AxioImager M2 confocal microscope with settings for bright field. The images were analyzed with AxioVision Rel 4.8 software and assembled with Photoshop Imaging Suite.

## Results

### *Hairless* encodes the tomato homolog of SRA1

**Fig. 2.**
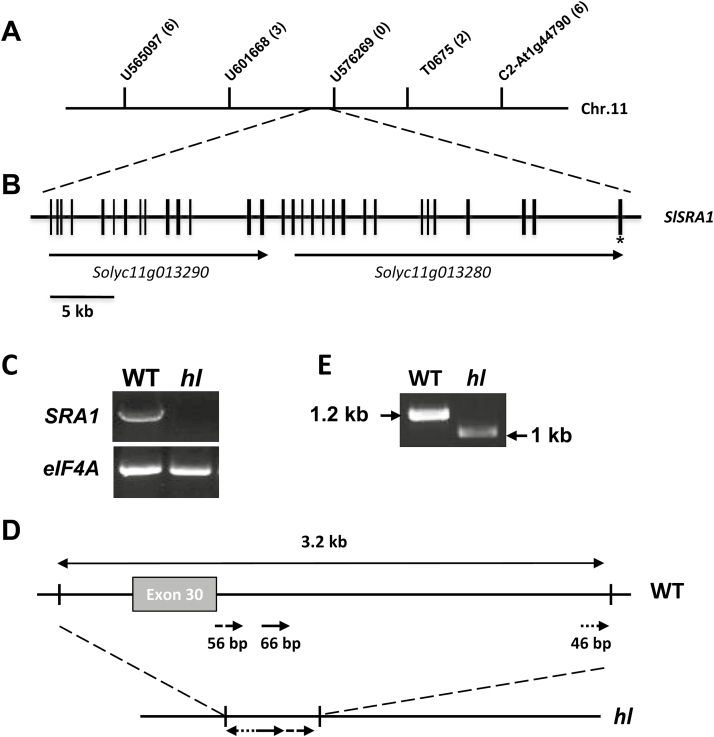
The *Hl* gene encodes the tomato homolog of *SRA1*. (A) Fine genetic mapping of *Hl* delimited the target gene to an interval between marker U601668 and T0675 on tomato chromosome 11. Numbers in parentheses indicate the number of recombination events identified between markers and the target gene. (B) Structure of *SlSRA1*. Vertical black lines depict exons and horizontal lines indicate intervening introns or intergenic regions. The mutation identified in the *hl* mutant affects the last exon, denoted with an asterisk. The relative location and direction of transcription of annotated genes (*Solyc11g013290* and *Solyc11g013280*) is shown. (C) Agarose gel showing the results of RT-PCR amplification of *SRA1* cDNAs using mRNA isolated from WT and *hl* leaves*. Elongation factor 4A* (*elF4A*) mRNA was used as a loading control. (D) Diagram depicting the nature of the deletion mutation identified in the *hl* mutant, as compared to the same genomic region of the WT. The *hl* mutation corresponds to a 3.2-kb deletion spanning the last exon of the gene. Arrows depict the directionality of short segments of DNA that are retained in the mutant. (E) Agarose gel showing the amplification products from 3ʹRACE of the *SRA1* cDNA, using mRNA isolated from WT and *hl* leaves.

The conspicuous absence of normal type I trichomes on leaves, stems, and hypocotyls of the *hl* mutant (Fig. 1) provided a robust morphological phenotype for fine mapping of the *Hl* locus, which was previously mapped to the short arm of chromosome 11 (Rick, 1980; Tanksley *et al.*, 1992). We used an F_2_ population derived from a cross between *hl* (LA3556) and *S. pennellii* (LA0716) to refine the map position of *Hl* to a ~300-kb region flanked by markers U601668 and T0675 (Fig. 2A). Among the predicted genes in this interval, proteins encoded by two adjacent loci (Solyc11g013280 and Solyc11g013290) were annotated as being homologous to the family of Cytoplasmic FMR1 (Fragile X mental retardation1) interacting proteins (CYFIPs), also known as PIR121 (P53-inducible protein 121) or SRA1 (Fig. 2B). The CYFIP/PIR121/SRA1 protein (hereafter referred to as SRA1) is a highly conserved component of the heteropentameric WRC that controls actin cytoskeletal dynamics by stimulating the activity of the ubiquitous ARP2/3 complex. In Arabidopsis, mutations in *SRA1* (At5g18410, also known as *KLUNKER*, *PIRP*, and *PIROGI*) or other components of the WRC result in distorted trichome phenotypes very similar to those observed in the tomato *hl* mutant (Basu *et al.*, 2004; Brembu *et al.*, 2004; Li *et al.*, 2004*b*; Saedler *et al.*, 2004). We thus considered Solyc11g013280 and Solyc11g013290 to be strong candidates for *Hl*.

Sequence alignments showed that Solyc11g013280 and Solyc11g013290 encode predicted proteins that correspond to the C- and N-terminal regions, respectively, of SRA1 in Arabidopsis and other species. This observation indicated that Solyc11g013280 and Solyc11g013290 might comprise a single locus. A ~42kb genomic region encompassing these two loci contains 30 annotated exons that specify a single coding region (Fig. 2B), which has an intron-exon organization that closely matches that of *SRA1* in Arabidopsis. To test whether Solyc11g013280 and Solyc11g013290 comprise a single gene, we performed RT-PCR with wild-type tomato leaf RNA and primers that span the putative ATG start codon in Solyc11g013290 and the TGA stop codon in Solyc11g013280. A single 3.86-kb transcript was amplified and sequenced (Fig. 2C). The cDNA is predicted to encode a 1287 amino-acid polypeptide that shares 81% identity with AtSRA1 (Supplementary Fig. S1). These findings show that Solyc11g013280 and Solyc11g013290 encode a single gene that corresponds to the tomato homolog of *SRA1*.

RT-PCR reactions performed with the same *SlSRA1* cDNA primer set and RNA from *hl* leaves failed to amplify a product (Fig. 2C). Sequencing of the entire 42-kb genomic region of *SRA1* in WT and *hl* plants showed that the mutant harbors a ~3-kb deletion encompassing the entire last (30th) exon within the coding region of the gene, a portion of the preceding intron, and the 3ʹ-untranslated region of the gene (Fig. 2D). Genomic DNA from *hl* retained interspersed sequences derived from the 3ʹ-UTR of WT DNA, suggesting that the deletion is associated with a more complex DNA rearrangement (Fig. 2D; Supplementary Fig. S2). To confirm the sequencing results and further test whether *SRA1* transcripts are expressed in *hl*, we used 3ʹRACE to amplify the affected region of the transcript. Sequencing of the resulting PCR product from WT and *hl* confirmed that the mutant produces an *SRA1* transcript that is truncated at the 3ʹ end (Fig. 2E). The mutant form of the transcript is predicted to encode a protein that lacks 63 amino acids at the C-terminus of SRA1 (Supplementary Fig. S3). These collective results demonstrate that the *hl* mutant harbors a deletion in the *SRA1* gene.

### Genetic complementation of the *hl* mutant restores normal trichome development

**Fig. 3.**
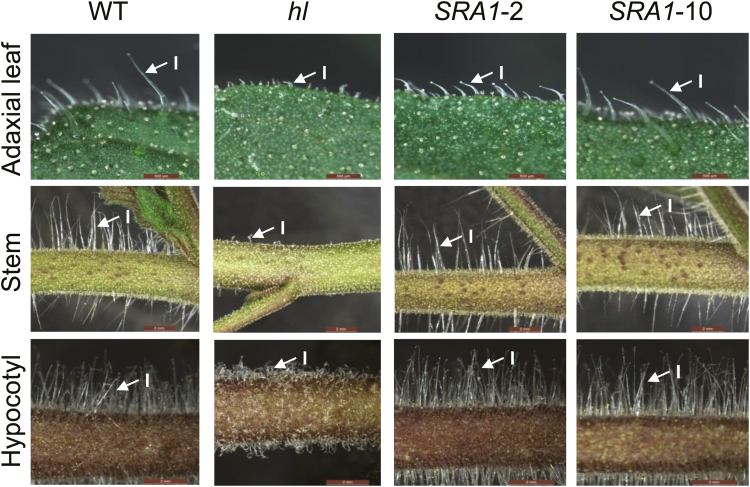
*SRA1* complements the trichome morphology defect of the *hl* mutant. Light microscopy was used to assess the trichome morphology of WT, *hl*, and two complemented transgenic lines (*SRA1*-2 and *SRA1*-10). Photographs show the adaxial leaf surface (upper row), stem (middle row), and hypocotyl (lower row) of each genotype. Arrows indicate representative type I trichomes. Scale bars: 500 µm, adaxial leaf surface; 2mm, stems and hypocotyls. Three-week-old plants were used for all photographs. (This figure is available in color at *JXB* online.)

To determine whether the mutation in *SRA1* causes the distorted trichome phenotype of *hl* plants, we used *Agrobacterium*-mediated transformation to complement the mutant with a cDNA that specifies the entire WT *SlSRA1* coding region, under the control of the *35S* promoter of *Cauliflower Mosaic Virus* (CaMV). Preliminary visual observations indicated that among seven independent primary (T_0_) transformants obtained, all exhibited normal trichome development on stems and hypocotyls (data not shown). Two of these lines (*SRA1*-2 and *SRA1*-10) segregated ~3:1 for the normal vs. distorted trichome phenotypes in the T_1_ generation and were selected for identification of corresponding T_2_ lines that are homozygous for the *35S:SRA1* transgene; these lines were used for all subsequent analyses. Light microscopy showed that the distorted trichome morphology on leaves, stems, and hypocotyls of the *hl* mutant was complemented by the *35S:SRA1* transgene in the *SRA1*-2 and *SRA1*-10 lines, which were indistinguishable from WT plants at this level of morphological analysis (Fig. 3). We also investigated the potential contribution of *SRA1* to trichome density, as it was previously reported that *hl* leaves have a modest reduction in the density of glandular trichomes (Kang *et al.* 2010*b*). We confirmed that the density of type VI trichomes on *hl* leaves was significantly less than that of WT leaves at the same developmental stage, and also found that this phenotype was complemented in the *SRA1*-2 and *SRA1*-10 transgenic lines (Supplementary Fig. S4). These data indicate that the defects in trichome morphology in the *hl* mutant result from a defective *SRA1* gene.

### *SRA1* modulates the production of trichome-borne metabolites and enhances resistance to insect herbivores

**Fig. 4.**
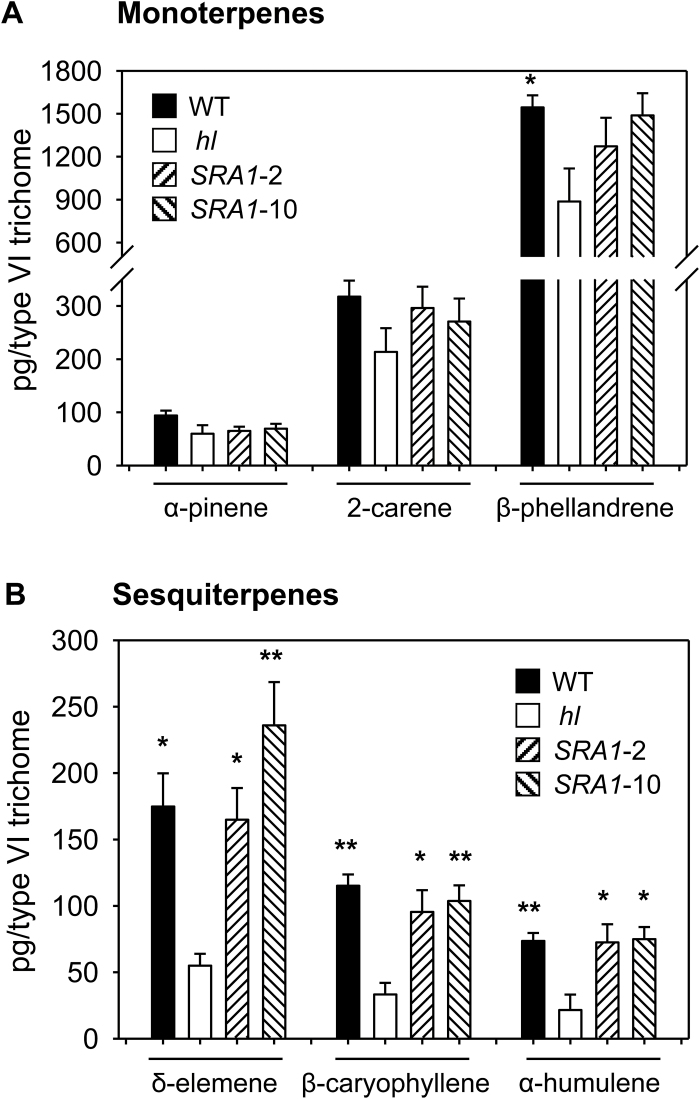
Mutation of *SRA1* affects terpenoid accumulation in type VI glands. Levels of monoterpenes (A) and sesquiterpenes (B) were measured in type VI glands isolated from leaves of WT, *hl*, and *SRA1*-complemented lines. Peak areas for each compound were normalized on a per gland basis. Each data point represents the mean ±SE of three or four independent plants per line. Asterisks represent significant differences between *hl* and other genotypes (unpaired *t*-test: *, *P*<0.05; **, *P*<0.01).

**Fig. 5.**
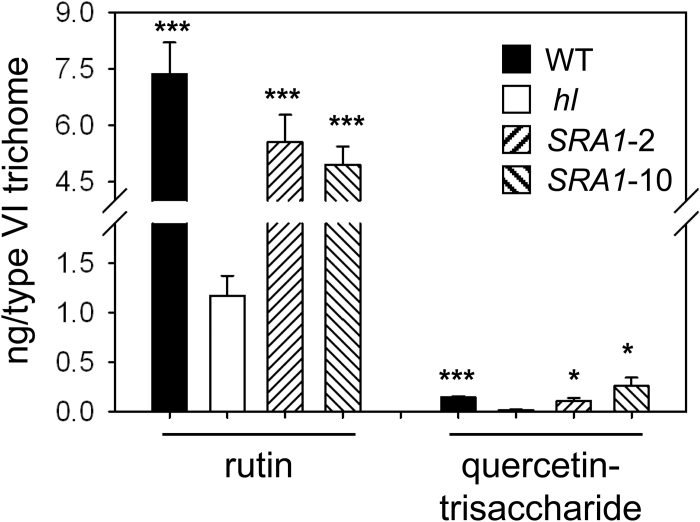
*SRA1* complements the flavonoid deficiency in type VI trichomes of *hl*. Rutin and quercetin-trisaccharide levels were measured in type VI glands isolated from leaves of WT, *hl*, and *SRA1*-complemented lines. Peak areas for each compound were normalized on a per gland basis. Each data point represents the mean ±SE of three or four independent plants per line. Asterisks represent significant differences between *hl* and other genotype plants (unpaired *t*-test: *, *P*<0.05; ***, *P*<0.001).

Previous studies showed that type VI glands on the *hl* mutant are deficient in the accumulation of various terpenoid and flavonoid derivatives that are implicated in plant anti-insect defense (Kang *et al.*, 2010*b*). To test the potential role of *SRA1* in the production of these compounds, we compared the level of terpenoids and flavonoids in type VI glands from WT, *hl*, and the two *SRA1*-complemented transgenic lines. Consistent with previous results, we found that isolated type VI glands from *hl* plants have significantly reduced levels of sesquiterpenes, whereas the monoterpene content in the mutant is unaffected (Fig. 4). The deficiency in sesquiterpene production in *hl* glands was fully complemented in both the *SRA1*-2 and *SRA1*-10 lines (Fig. 4). We also found that the reduced capacity of type VI glands from *hl* leaves to produce rutin and quercetin trisaccaride was restored to WT levels in the *SRA1*-2 and *SRA1*-10 plants (Fig. 5). These results establish a role for *SRA1* in the production of sesquiterpene and flavonoid derivatives in type VI glandular trichomes.

**Fig. 6.**
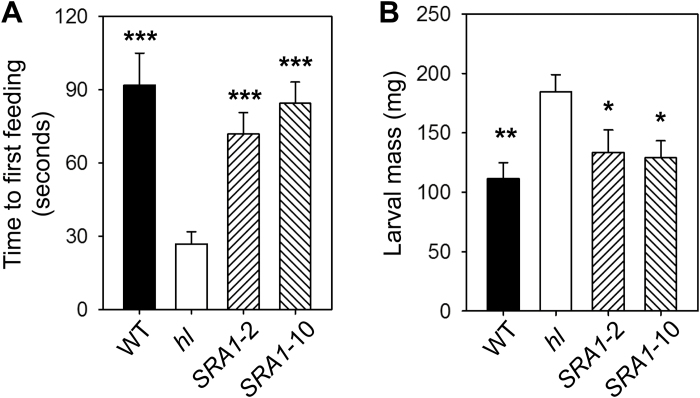
*SRA1* restores resistance of *hl* plants to feeding by *Manduca sexta* larvae. (A) Newly hatched larvae were placed on the adaxial leaf surface of the indicated genotype, and the time to first feeding on the leaf lamina was measured. Data show the mean (±SE) time to first feeding for nine *M. sexta* larvae on leaves of WT, *hl*, and two *SRA1*-complemented lines. (B) No choice bioassays were performed by placing two newly hatched *M. sexta* larvae on leaves of each plant genotype. Data show the mean (±SE) mass of larvae (*n*=28) reared for 8 d on the plant. Asterisks represent significant differences between *hl* and other genotypes (unpaired *t*-test: *, *P*<0.05; **, *P*<0.01; ***, *P*<0.001).

The effect of *hl* on the morphology and chemical composition of leaf trichomes suggested that the mutation might be responsible for the increased susceptibility of the mutant to insect herbivores. To address this question, WT, *hl*, and *SRA1*-complemented plants were challenged with newly hatched *M. sexta* larvae. Insect behavior and time-to-first feeding were observed immediately after the challenge. In comparison to insects on WT plants, larvae that were placed onto *hl* leaves took significantly less time to begin feeding on the leaf lamina (Fig. 6A). Observation of larval behavior suggested that this effect resulted, at least in part, from the presence of type I trichomes that initially impeded the ability of larvae to access feeding sites on the leaf lamina. The feeding behavior of insect larvae on the *SRA1*-complemented lines was indistinguishable from that of insects on WT plants. Following 8 d of continuous feeding, larvae grown on *hl* plants were significantly heavier than those reared on either WT or the transgenic complemented lines (Fig. 6B). These results demonstrate that *SRA1* enhances the resistance of tomato to insect herbivores.

### *SRA1* affects the mechanical and cell anatomical properties of stem tissue

**Fig. 7.**
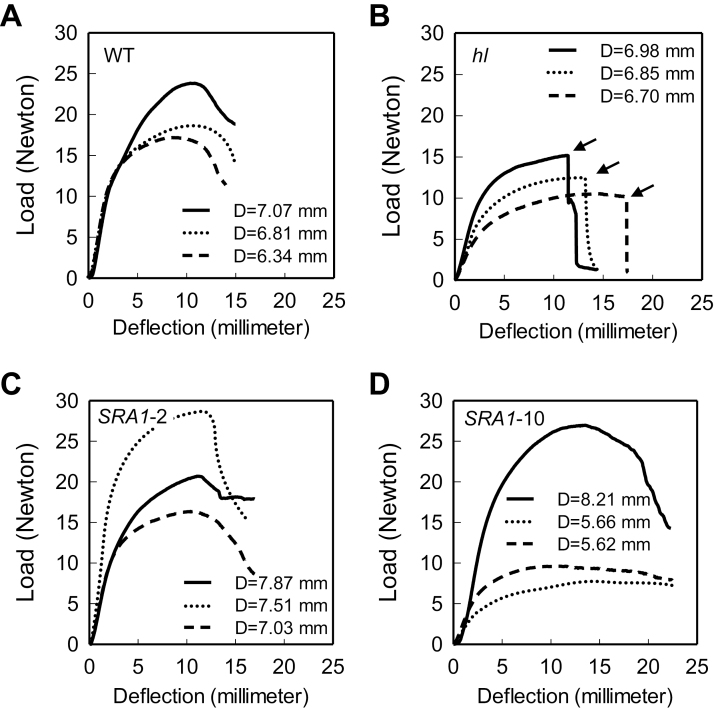
*SRA1* complements the brittleness of *hl* stem tissue. Stem segments from WT (A), *hl* (B) and two transgenic lines, *SRA1*-2 (C) and *SRA1*-10 (D), were subject to a four-point bending test as described in ‘Materials and Methods’. The three traces in each panel correspond the three biological replicates per genotype. The average diameter, D, of each stem segment is indicated within the graph. The arrows in panel B indicate the point of complete failure of *hl* stems.

In addition to effects on trichome morphology, *hl* was previously described as having brittle stems (Butler, 1952; Dempsey and Sherif, 1987). In preliminary experiments we confirmed this trait by observing the behavior of hand-bent stem segments. Bending of WT stems invariably resulted in buckling but not breaking or snapping of the stem (Supplementary Video S1). In contrast, bending of *hl* stems caused the tissue to snap rather than buckle (Supplementary Video S2). Tests performed with *SRA1*-2 and *SRA1*-10 showed that stems from these lines, like that of WT, buckled but did not break or snap (Supplementary Videos S3, S4). Next, we conducted four-point bending tests to quantify the contribution of *SRA1* to the material properties of stem tissue. WT stems showed a typical response curve of a ductile composite material while lacking a distinct point of failure (Fig. 7A). However, the response of *hl* stems was characterized by an instant and complete stem failure, reminiscent of brittle materials (Fig. 7B). Loading curves obtained for both *SRA1*-complemented lines were also similar to WT plants (Fig. 7C, D). No significant differences in the elastic modulus were observed between the WT, *hl* and *SRA1*-complemented lines. However, a significant reduction in stem strength (modulus of rupture) was observed in the *hl* line compared to WT, and this reduction in strength was restored in the *SRA1*-complemented lines (data not shown). These results indicate that the brittleness and reduced strength of *hl* mutant results from a defect in the *SRA1* gene.

**Fig. 8.**
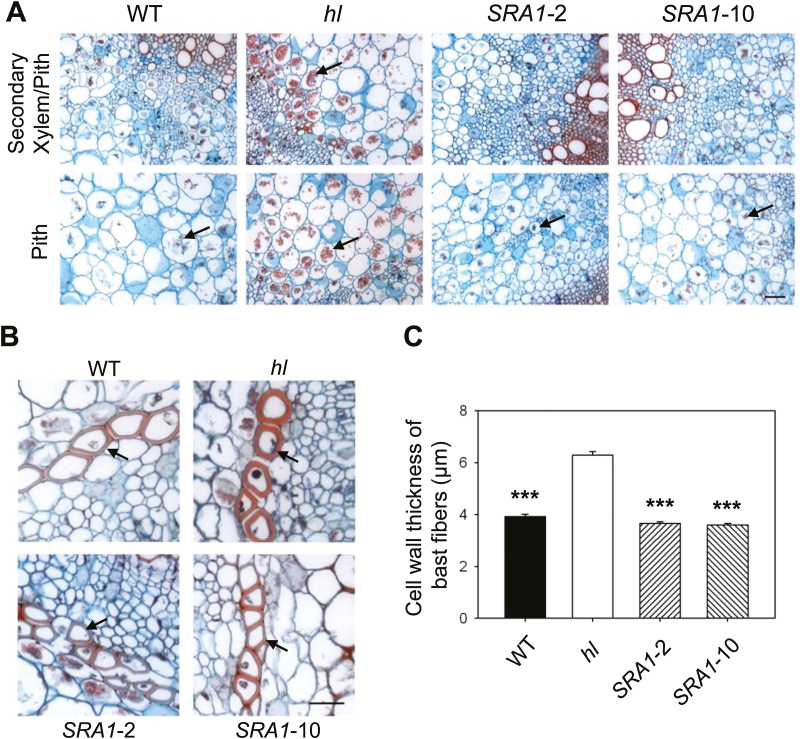
Mutation of *SRA1* in tomato affects cell anatomy of stem tissue. Cross sections of stem tissue from WT, *hl*, and *SRA1*-complemented lines (*SRA1*-2 and *SRA1*-10) were stained with Safranin O and Fast Green. (A) Cells within the secondary xylem and pith regions of *hl* contain red-colored granular structures (arrows) that are less prevalent in WT and *SRA1*-complemented lines. (B) Bast fibers of *hl* stems have thicker cell walls (arrows) than those of WT and *SRA1*-complemented line stems. (C) Quantification of cell wall thickness of bast fibers. Data show the mean (±SE) cell wall thickness of at least 60 of bast fibers from three biological replicates per genotype. Asterisks represent significant differences between *hl* and other genotypes (unpaired *t*-test: ***, *P*<0.001). Scale bars: 100 µm (A); 50 µm (B).

To determine whether the brittleness of *hl* stems is associated with changes in cell and tissue anatomy, we performed histological analyses to identify lignin-rich and cellulose-rich regions of the stem. The morphology of vascular bundle and pith cells was very similar between WT and *hl* stems (Fig. 8A). Pith cells of *hl* stems, however, were characterized by the presence of granular material that was much less conspicuous in stem sections from WT and the *SRA1*-complemented lines (Fig. 8A). Positive staining of these granules with iodine suggested the presence of starch or related compounds (Supplementary Fig. S5). We also found that the secondary cell walls of bast fiber cells of *hl* mutant were significantly thicker than those of WT and the complemented lines (Fig. 8B, C). These results indicate that defects in *SRA1* expression affect cell homeostasis and stem tensile strength.

## Discussion

### *Hl* encodes the tomato homolog of SRA1

Mutational analysis of unicellular trichome development in Arabidopsis has been instrumental for deciphering mechanisms of plant cell fate determination and differentiation. Experimental studies with actin inhibitors and the ‘distorted group’ of trichome mutants have been particularly useful in establishing a role for actin filament organization in polarized cell growth and cell shape (Hulskamp *et al.*, 1994; Mathur *et al.*, 1999; Szymanski *et al.*, 1999; Yanagisawa *et al.*, 2013). Molecular studies have revealed that genes defined by a subset of trichome distortion mutants encode subunits of the ARP2/3 actin filament nucleation complex, as well as components of the WRC that controls the actin-nucleating activity of ARP2/3 complexes (Le *et al.*, 2003; El-Assal *et al.*, 2004*a*, *b*; Szymanski, 2005). Based on the phenotypic similarity (e.g. severe bending and swelling of trichomes) between these mutants and the tomato *hl* mutant, it was previously suggested that *Hl* might perform a functional role in the actin cytoskeleton network (Kang *et al.*, 2010*b*). Here, we provide molecular genetic evidence to validate this hypothesis.

Our map-based cloning studies localized the *hl* mutation to the tomato homolog of *SRA1*. In animals, SRA1 is a subunit of the heteropentameric WRC and is essential for proper control of actin filament assembly (Eden *et al.*, 2002). The WRC is activated by a multitude of signals at the plasma membrane, including kinases, acidic phospholipids, and the small GTPase Rac. Recent studies in both animal and plant systems have identified receptors that link the WRC to distinct membrane sites, thus establishing a mechanism to promote actin filament assembly in a highly specific, polarized manner (Chen *et al.*, 2014; Facette *et al.*, 2015). The central role of SRA1 in actin organization is highlighted by its ability to link the WRC, via direct protein-protein interaction, to upstream effectors (e.g. Rac). Given that *sra1* mutants of Arabidopsis display cell shape and actin-related phenotypes that are very similar to those of ARP2/3 complex mutants, it is generally thought that the WRC promotes ARP2/3 complex activity in plant cells (Mathur *et al.*, 2003; El-Assall *et al.*, 2004*a*, *b*; Li *et al.*, 2004*b*). In support of this view, the Arabidopsis and human homologs of SRA1, which share ~30% amino acid identity, are functionally interchangeable (Basu *et al.*, 2004).

The trichome distortion phenotype of *hl* plants is similar to that of *sra1* null mutations in Arabidopsis (Basu *et al.*, 2004; Brembu *et al.*, 2004; Li *et al.*, 2004*b*; Saedler *et al.*, 2004). Given the established role of SRA1 in actin dynamics, our results strongly indicate that the trichome morphological phenotypes of the *hl* mutant result from perturbation of the actin-cytoskeleton network. We note, however, that the wide range of developmental and growth phenotypes exhibited by *sra1* mutants of Arabidopsis, which include leaf epinasty, defects in epidermal cell-cell adhesion, reduced chlorophyll content, and reproductive abnormalities (Li *et al.*, 2004*b*), are not apparent in the tomato *hl* mutant under our growth conditions (Kang *et al.*, 2010*b*; this study). These observations raise the question of whether *hl* is a null allele of *SRA1* or whether the mutant produces a truncated protein that retains partial activity. The *hl* mutation is predicted to generate a C-terminally truncated form of SRA1 lacking several amino acid residues that are strictly conserved between plants, flies, and humans. Given that we detected truncated *SRA1* transcripts in *hl* tissues, additional studies are needed to more precisely determine how the *hl* mutation affects SRA1 function.

### Role of SRA1 in trichome chemistry and function

Previously characterized trichome mutants of cultivated tomato, including *af* and *od-2*, have type VI trichomes that are broadly deficient in the accumulation of monoterpenes, sesquiterpenes, and flavonoid derivatives (Kang *et al.*, 2010*a*, 2014). Interestingly, however, type VI trichomes of the *hl* mutant accumulate monoterpenes but not sesquiterpenes or flavonoids (Kang *et al.*, 2010*b*; this study). One obvious difference between these broad metabolite classes is their biosynthetic location; whereas monoterpene biosynthesis occurs in plastids, enzymes involved in sesquiterpene and flavonoid production are, in general, located in the cytosol. Our results thus raise the possibility that flavonoid and sesquiterpenoid biosynthesis in the cytosol of glandular trichomes is dependent on the actin-cytoskeleton network. It is conceivable that the cytoskeleton provides a scaffold on which to organize biosynthetic enzymes or facilitate transport of metabolic end products to the vacuole (in the case of flavonoids) or other cellular locations for storage (Widhalm *et al.*, 2015). Indeed, there is considerable evidence implicating the actin cytoskeleton in plant immune responses, including intracellular trafficking of vesicles (Day *et al.*, 2011). Given that SRA1 also controls translation initiation in animal cells (Schenck *et al.*, 2003; Napoli *et al.*, 2008), we cannot exclude the possibility that changes in chemical composition of *hl* trichomes result from defects in cellular processes other than actin assembly dynamics.

Cultivated tomato uses a multi-layered defense system for protection against a broad spectrum of arthropod herbivores (Howe and Jander, 2008; Peiffer *et al.*, 2009; Campos *et al.*, 2014). Wound- and jasmonate-inducible proteinase inhibitors, amino acid degrading enzymes, and secondary metabolites comprise the dominant chemical defense system within the tomato leaf lamina (Green and Ryan, 1973; Chen *et al.*, 2005, 2006; Gonzales-Vigil *et al.*, 2011). As epidermal structures, trichomes provide constitutive physical and chemical barriers that protect underlying leaf lamina tissues from herbivores. Several lines of evidence indicate that the susceptibility of the *hl* mutant to insect herbivores can be attributed to a defect in trichome-mediated resistance (Kang *et al.*, 2010*b*; Tian *et al.*, 2012). First, wound-induced production of leaf proteinase inhibitors is not impaired in the *hl* mutant (Kang *et al.*, 2010*b*). Second, the *hl* mutant exhibits abnormal trichome development and also accumulates low levels of several trichome-borne metabolites, including flavonoids (e.g. rutin) and sesquiterpenoids, that have been implicated in anti-insect defense (Kennedy, 2003; Kang *et al.*, 2010*b*). Finally, transgenic complementation studies demonstrate that loss of resistance to the tomato specialist *M. sexta*, as well as defects in trichome morphology and chemistry, is caused by mutation of *SRA1*. Although the results of our insect bioassays clearly show that *SRA1* contributes to host resistance, additional studies are needed to determine the extent to which loss of SRA1 comprises either physical or chemical aspects of trichome resistance. It is also possible that *hl* impairs the role of glandular trichomes as sensors to detect movement of insect herbivores on the leaf surface (Peiffer *et al.*, 2009).

### Role of SRA1 in mechanical properties of stem tissue

In addition to trichome-related phenotypes, stems of *hl* mutant plants exhibit increased fragility. Dempsey and Sherif (1987) initially noted that the increased brittleness of *hl* stems is associated with irregularities in phloem fibers of vascular bundles. They also reported that cellulosic microfibrils appear disorganized in the mutant, and that the vesicle content within the cytoplasm is altered as well. Our finding that the *hl* defines a key regulator (SRA1) of actin filament organization provides a new insight into the molecular basis of stem fragility in the mutant. Primary and secondary cell walls have a major impact on the strength and mechanical properties of plant tissues (Niklas and Spatz, 2012). An increasing body of molecular evidence links the cytoskeleton network to various processes involved in the synthesis and deposition of cell wall polymers, which are required for normal cell morphogenesis. Molecular genetic analysis of the *fragile fiber* class of mutants in Arabidopsis, for example, has uncovered several genes involved in the organization of actin and cortical microtubules, including genes required for the trafficking of vesicles that contain cell wall components (Zhong *et al.*, 2004; Bannigan and Baskin, 2005). *fra3* mutations cause a dramatic reduction in secondary wall thickness and a concomitant decrease in stem strength (Zhong *et al.*, 2004). In rice, genetic analysis of the *brittle culm* group of mutants has similarly shown that cell wall synthesis and remodeling are critical for the mechanical strength of tissues (Zhang and Zhou, 2011). Based on the association between the mechanical properties of *hl* stems and altered histological features of vascular bundle and pith cells (Dempsey and Sherif, 1987; this study), it is possible that SRA1 is required for actin cytoskeleton-dependent processes that direct the synthesis or proper deposition of cell wall components, and that disruption of this process in *hl* alters the mechanical properties of stem tissue. It is well established that the WRC responds to upstream input signals to spatially and temporally regulate actin filament assembly to direct vesicle trafficking (Takenawa and Suetsugu, 2007; Yanagisawa *et al.*, 2013). Our results thus provide a foundation on which to better understand how the actin-cytoskeleton network directs the assembly of cell wall polymers that determine mechanical properties of plant tissues.

## Supplementary data

Supplementary data are available at *JXB* online.

Table S1. Description of PCR-based mapping markers.

Table S2. Description of PCR primers used in this study.

Figure S1. Amino acid sequence comparison of tomato and Arabidopsis SRA1.

Figure S2. Genomic sequence of the affected region in WT and *hl* plants.

Figure S3. Comparison of the predicted amino acid sequence of SRA1 in WT and *hl* plants.

Figure S4. Density of type VI trichomes on leaves from WT, *hl*, and *SRA1*-complemented transgenic lines.

Figure S5. Iodine staining of pith cells from WT and *hl* stems.

Video S1. Manual bending test of WT stem.

Video S2. Manual bending test of *hl* stem.

Video S3. Manual bending test of *SRA1*-2 stem.

Video S4. Manual bending test of *SRA1*-10 stem.

Supplementary Data

## References

[CIT0001] Bannigan A Baskin TI 2005 Directional cell expansion – turning toward actin. Current Opinion in Plant Biology 8, 619–624. 16181803 10.1016/j.pbi.2005.09.002

[CIT0002] Basu D El-Assal SED Le J Mallery EL Szymanski DB 2004 Interchangeable functions of Arabidopsis PIROGI and the human WAVE complex subunit SRA1 during leaf epidermal development. Development 131, 4345–4355. 15294869 10.1242/dev.01307

[CIT0003] Bergau N Bennewitz S Syrowatka F Hause G Tissier A 2015 The development of type VI glandular trichomes in the cultivated tomato *Solanum lycopersicum* and a related wild species *S. habrochaites* . BMC Plant Biology 15, 289. 26654876 10.1186/s12870-015-0678-z PMC4676884

[CIT0004] Brembu T Winge P Seem M Bones AM 2004 *NAPP* and *PIRP* encode subunits of a putative wave regulatory protein complex involved in plant cell morphogenesis. The Plant Cell 16, 2335–2349. 15316111 10.1105/tpc.104.023739 PMC520937

[CIT0005] Butler L 1952 The linkage map of the tomato. Journal of Heredity 43, 25–35.

[CIT0006] Campos ML Kang JH Howe GA 2014 Jasmonate-triggered plant immunity. Journal of Chemical Ecology 40, 657–675. 24973116 10.1007/s10886-014-0468-3 PMC4143990

[CIT0007] Chen BY Brinkmann K Chen ZC Pak CW Liao YX Shi SY Henry L Grishin NV Bogdan S Rosen MK 2014 The WAVE regulatory complex links diverse receptors to the actin cytoskeleton. Cell 156, 195–207. 24439376 10.1016/j.cell.2013.11.048 PMC4059610

[CIT0008] Chen H Wilkerson CG Kuchar JA Phinney BS Howe GA 2005 Jasmonate-inducible plant enzymes degrade essential amino acids in the herbivore midgut. Proceedings of the National Academy of Sciences, USA 102, 19237–19242. 10.1073/pnas.0509026102 PMC1323180 16357201

[CIT0009] Chen H Jones AD Howe GA 2006 Constitutive activation of the jasmonate signaling pathway enhances the production of secondary metabolites in tomato. FEBS Letters 580, 2540–2546. 16647069 10.1016/j.febslet.2006.03.070

[CIT0010] Day B Henty JL Porter KJ Staiger CJ 2011 The pathogen-actin connection: a platform for defense signaling in plants. Annual Review of Phytopathology 49, 483–506. 10.1146/annurev-phyto-072910-095426 21495845

[CIT0011] Dempsey WH Sherif THI 1987 Brittleness in the stem of the seven ‘hairless’ mutants. Report of the Tomato Genetics Cooperative 37, 4.

[CIT0012] Eden S Rohatgi R Podtelejnikov AV Mann M Kirschner MW 2002 Mechanism of regulation of WAVE1-induced actin nucleation by Rac1 and Nck. Nature 418, 790–793. 12181570 10.1038/nature00859

[CIT0013] El-Assal SED Le J Basu D Mallery EL Szymanski DB 2004 *a* Arabidopsis *GNARLED* encodes a NAP125 homolog that positively regulates ARP2/3. Current Biology 14, 1405–1409. 15296760 10.1016/j.cub.2004.06.062

[CIT0014] El-Assall SE Le J Basu D Mallery EL Szymanski DB 2004 *b* DISTORTED2 encodes an ARPC2 subunit of the putative Arabidopsis ARP2/3 complex. The Plant Journal 38, 526–538. 15086808 10.1111/j.1365-313X.2004.02065.x

[CIT0015] Facette MR Park Y Sutimantanapi D Luo AD Cartwright HN Yang B Bennett EJ Sylvester AW Smith LG 2015 The SCAR/WAVE complex polarizes PAN receptors and promotes division asymmetry in maize. Nature Plants 1, article number: 14024. 10.1038/nplants.2014.24 27246760

[CIT0016] Goffreda JC Szymkowiak EJ Sussex IM Mutschler MA 1990 Chimeric tomato plants show that aphid resistance and triacylglucose production are epidermal autonomous characters. The Plant Cell 2, 643–649. 2136638 10.1105/tpc.2.7.643 PMC159918

[CIT0017] Gonzales-Vigil E Bianchetti CM Phillips GN Howe GA 2011 Adaptive evolution of threonine deaminase in plant defense against insect herbivores. Proceedings of the National Academy of Sciences, USA 108, 5897–5902. 10.1073/pnas.1016157108 PMC3078374 21436043

[CIT0018] Green TR Ryan CA 1973 Wound-induced proteinase inhibitor in plant leaves: a possible defense mechanism against insects. Science 175, 776–777. 17836138 10.1126/science.175.4023.776

[CIT0019] Howe G Jander G 2008 Plant immunity to insect herbivores. Annual Review of Plant Biology 59, 41–66. 10.1146/annurev.arplant.59.032607.092825 18031220

[CIT0020] Hulskamp M Misra S Jurgens G 1994 Genetic dissection of trichome cell development in Arabidopsis. Cell 76, 555–566. 8313475 10.1016/0092-8674(94)90118-x

[CIT0021] Johansen D 1940 Plant Microtechnique. McGraw Hill: London, UK.

[CIT0022] Kang JH Liu GH Shi F Jones AD Beaudry RM Howe GA 2010 *a* The tomato *odorless-2* mutant is defective in trichome-based production of diverse specialized metabolites and broad-spectrum resistance to insect herbivores. Plant Physiology 154, 262–272. 20668059 10.1104/pp.110.160192 PMC2938144

[CIT0023] Kang JH McRoberts J Shi F Moreno JE Jones AD Howe GA 2014 The flavonoid biosynthetic enzyme chalcone isomerase modulates terpenoid production in glandular trichomes of tomato. Plant Physiology 164, 1161–1174. 24424324 10.1104/pp.113.233395 PMC3938611

[CIT0024] Kang JH Shi F Jones AD Marks MD Howe GA 2010 *b* Distortion of trichome morphology by the *hairless* mutation of tomato affects leaf surface chemistry. Journal of Experimental Botany 61, 1053–1064. 20018901 10.1093/jxb/erp370 PMC2826649

[CIT0025] Karabourniotis G Papadopoulos K Papamarkou M Manetas Y 1992 Ultraviolet-B radiation absorbing capacity of leaf hairs. Physiologia Plantarum 86, 414–418.

[CIT0026] Karlgren A Carlsson J Gyllenstrand N Lagercrantz U Sundstrom JF 2009 Non-radioactive *in situ* hybridization protocol applicable for Norway spruce and a range of plant species. Journal of Visualized Experiments, e1205. 10.3791/1205 PMC3148633 19377440

[CIT0027] Katsir L Schilmiller AL Staswick PE He SY Howe GA 2008 COI1 is a critical component of a receptor for jasmonate and the bacterial virulence factor coronatine. Proceedings of the National Academy of Sciences, USA 105, 7100–7105. 10.1073/pnas.0802332105 PMC2383947 18458331

[CIT0028] Kennedy GG 2003 Tomato, pests, parasitoids, and predators: Tritrophic interactions involving the genus Lycopersicon. Annual Review of Entomology 48, 51–72. 10.1146/annurev.ento.48.091801.112733 12194909

[CIT0029] Koo AJK Chung HS Kobayashi Y Howe GA 2006 Identification of a peroxisomal acyl-activating enzyme involved in the biosynthesis of jasmonic acid in Arabidopsis. Journal of Biological Chemistry 281, 33511–33520. 16963437 10.1074/jbc.M607854200

[CIT0030] Le J El-Assal Sel D Basu D Saad ME Szymanski DB 2003 Requirements for Arabidopsis ATARP2 and ATARP3 during epidermal development. Current Biology 13, 1341–1347. 12906796 10.1016/s0960-9822(03)00493-7

[CIT0031] Li C Liu G Xu C Lee GI Bauer P Ling HQ Ganal MW Howe GA 2003 The tomato suppressor of prosystemin-mediated responses2 gene encodes a fatty acid desaturase required for the biosynthesis of jasmonic acid and the production of a systemic wound signal for defense gene expression. The Plant Cell 15, 1646–1661. 12837953 10.1105/tpc.012237 PMC165407

[CIT0032] Li L Howe GA 2001 Alternative splicing of prosystemin pre-mRNA produces two isoforms that are active as signals in the wound response pathway. Plant Molecular Biology 46, 409–419. 11485198 10.1023/a:1010645330275

[CIT0033] Li L Zhao Y McCaig BC Wingerd BA Wang J Whalon ME Pichersky E Howe GA 2004 *a* The tomato homolog of CORONATINE-INSENSITIVE1 is required for the maternal control of seed maturation, jasmonate-signaled defense responses, and glandular trichome development. The Plant Cell 16, 126–143. 14688297 10.1105/tpc.017954 PMC301400

[CIT0034] Li Y Sorefan K Hemmann G Bevan MW 2004 *b* Arabidopsis NAP and PIR regulate actin-based cell morphogenesis and multiple developmental processes. Plant Physiology 136, 3616–3627. 15516496 10.1104/pp.104.053173 PMC527160

[CIT0035] Liu Y Xu F Gou J Al-Haddad J Telewski FW Bae HJ Joshi CP 2012 Importance of two consecutive methionines at the N-terminus of a cellulose synthase (PtdCesA8A) for normal wood cellulose synthesis in aspen. Tree Physiology 32, 1403–1412. 23076823 10.1093/treephys/tps096

[CIT0036] Luckwill L 1943 The genus *Lycopersicon*: historical, biological, and taxonomic survey of the wild and cultivated tomatoes. Aberdeen University Press: Aberdeen, Scotland.

[CIT0037] Marks MD 1997 Molecular genetic analysis of trichome development in Arabidopsis. Annual Review of Plant Physiology and Plant Molecular Biology 48, 137–163. 10.1146/annurev.arplant.48.1.137 15012260

[CIT0038] Mathur J Chua NH 2000 Microtubule stabilization leads to growth reorientation in Arabidopsis trichomes. The Plant Cell 12, 465–477. 10760237 10.1105/tpc.12.4.465 PMC139846

[CIT0039] Mathur J Mathur N Kernebeck B Hulskamp M 2003 Mutations in actin-related proteins 2 and 3 affect cell shape development in Arabidopsis. The Plant Cell 15, 1632–1645. 12837952 10.1105/tpc.011676 PMC165406

[CIT0040] Mathur J Spielhofer P Kost B Chua N 1999 The actin cytoskeleton is required to elaborate and maintain spatial patterning during trichome cell morphogenesis in *Arabidopsis thaliana* . Development 126, 5559–5568. 10572033 10.1242/dev.126.24.5559

[CIT0041] McDowell ET Kapteyn J Schmidt A 2011 Comparative functional genomic analysis of Solanum glandular trichome types. Plant Physiology 155, 524–539. 21098679 10.1104/pp.110.167114 PMC3075747

[CIT0042] Napoli I Mercaldo V Boyl PP 2008 The fragile X syndrome protein represses activity-dependent translation through CYFIP1, a new 4E-BP. Cell 134, 1042–1054. 18805096 10.1016/j.cell.2008.07.031

[CIT0043] Niklas KJ 1992 Plant biomechanics: an engineering approach to plant form and function. University of Chicago Press: Chicago, USA.

[CIT0044] Niklas KJ Spatz H-C 2012 Plant Physics. University of Chicago Press: Chicago, USA.

[CIT0045] Paetzold H Garms S Bartram S 2010 The isogene 1-deoxy-D-xylulose 5-phosphate synthase 2 controls isoprenoid profiles, precursor pathway allocation, and density of tomato trichomes. Molecular Plant 3, 904–916. 20591838 10.1093/mp/ssq032

[CIT0046] Peiffer M Tooker JF Luthe DS Felton GW 2009 Plants on early alert: glandular trichomes as sensors for insect herbivores. New Phytologist 184, 644–656. 19703113 10.1111/j.1469-8137.2009.03002.x

[CIT0047] Pruyn ML Ewers IB Telewski FW 2000 Thigmomorphogenesis: changes in the morphology and mechanical properties of two Populus hybrids in response to mechanical perturbation. Tree Physiology 20, 535–540. 12651434 10.1093/treephys/20.8.535

[CIT0048] Ramsay NA Glover BJ 2005 MYB-bHLH-WD40 protein complex and the evolution of cellular diversity. Trends in Plant Science 10, 63–70. 15708343 10.1016/j.tplants.2004.12.011

[CIT0049] Reeves AF 1977 Tomato trichomes and mutations affecting their development. American Journal of Botany 64, 186–189.

[CIT0050] Rick CM 1980 Tomato linkage survey. Report of the Tomato Genetics Cooperative 30, 2–17.

[CIT0051] Rick CM Butler L 1956 Cytogenetics of the tomato. Advances in Genetics 8, 267–282.

[CIT0052] Rick CM Quiros CF Lange WH Stevens MA 1976 Monogenic control of resistance in tomato to tobacco flea beetle: probable repellence by floiar volatiles. Euphytica 25, 521–530.

[CIT0053] Saedler R Zimmermann I Mutondo M Hulskamp M 2004 The Arabidopsis *KLUNKER* gene controls cell shape changes and encodes the AtSRA1 homolog. Plant Molecular Biology 56, 775–782. 15803414 10.1007/s11103-004-4951-z

[CIT0054] Sallaud C Rontein D Onillon S 2009 A novel pathway for sesquiterpene biosynthesis from Z,Z-farnesyl pyrophosphate in the wild tomato *Solanum habrochaites* . The Plant Cell 21, 301–317. 19155349 10.1105/tpc.107.057885 PMC2648096

[CIT0055] Schenck A Bardoni B Langmann C Harden N Mandel JL Giangrande A 2003 CYFIP/Sra-1 controls neuronal connectivity in Drosophila and links the Rac1 GTPase pathway to the fragile X protein. Neuron 38, 887–898. 12818175 10.1016/s0896-6273(03)00354-4

[CIT0056] Schilmiller AL Charbonneau AL Last RL 2012 Identification of a BAHD acetyltransferase that produces protective acyl sugars in tomato trichomes. Proceedings of the National Academy of Sciences, USA 109, 16377–16382. 10.1073/pnas.1207906109 PMC3479610 22988115

[CIT0057] Schilmiller AL Koo AJK Howe GA 2007 Functional diversification of acyl-CoA oxidases in jasmonic acid biosynthesis and action. Plant Physiology 143, 812–824 17172287 10.1104/pp.106.092916 PMC1803733

[CIT0058] Schilmiller AL Last RL Pichersky E 2008 Harnessing plant trichome biochemistry for the production of useful compounds. The Plant Journal 54, 702–711. 18476873 10.1111/j.1365-313X.2008.03432.x

[CIT0059] Schilmiller AL Schauvinhold I Larson M Xu R Charbonneau AL Schmidt A Wilkerson C Last RL Pichersky E 2009 Monoterpenes in the glandular trichomes of tomato are synthesized from a neryl diphosphate precursor rather than geranyl diphosphate. Proceedings of the National Academy of Sciences, USA 106, 10865–10870. 10.1073/pnas.0904113106 PMC2705607 19487664

[CIT0060] Szymanski DB 2005 Breaking the WAVE complex: the point of Arabidopsis trichomes. Current Opinion in Plant Biology 8, 103–112. 15653407 10.1016/j.pbi.2004.11.004

[CIT0061] Szymanski DB Marks MD Wick SM 1999 Organized F-actin is essential for normal trichome morphogenesis in Arabidopsis. The Plant Cell 11, 2331–2347. 10590162 10.1105/tpc.11.12.2331 PMC144140

[CIT0062] Takenawa T Suetsugu S 2007 The WASP-WAVE protein network: connecting the membrane to the cytoskeleton. Nature Review Molecular and Cellular Biology 8, 37–48. 17183359 10.1038/nrm2069

[CIT0063] Tanksley SD Ganal MW Prince JP 1992 High-density molecular linkage maps of the tomato and potato genomes. Genetics 132, 1141–1160. 1360934 10.1093/genetics/132.4.1141 PMC1205235

[CIT0064] Tian D Tooker J Peiffer M Chung SH Felton GW 2012 Role of trichomes in defense against herbivores: comparison of herbivore response to *woolly* and *hairless* trichome mutants in tomato (*Solanum lycopersicum*). Planta 236, 1053–1066. 22552638 10.1007/s00425-012-1651-9

[CIT0065] Tomato Genome Consortium 2012 The tomato genome sequence provides insights into fleshy fruit evolution. Nature 485, 635–641. 22660326 10.1038/nature11119 PMC3378239

[CIT0066] Werker E 2000 Trichome diversity and development. Advances in botanical research incorporating advances. In: Plant Pathology 31, 1–35.

[CIT0067] Widhalm JR Jaini R Morgan JA Dudareva N 2015 Rethinking how volatiles are released from plant cells. Trends in Plant Science 20, 545–550. 26189793 10.1016/j.tplants.2015.06.009

[CIT0068] Yanagisawa M Zhang C Szymanski DB 2013 ARP2/3-dependent growth in the plant kingdom: SCARs for life. Frontiers in Plant Science 4, 166. 23802001 10.3389/fpls.2013.00166 PMC3689024

[CIT0069] Yang C Ye Z 2013 Trichomes as models for studying plant cell differentiation. Cellular and Molecular Life Sciences 70, 1937–1948. 22996257 10.1007/s00018-012-1147-6 PMC11113616

[CIT0070] Yang CX Li HX Zhang JH 2011 A regulatory gene induces trichome formation and embryo lethality in tomato. Proceedings of the National Academy of Sciences, USA 108, 11836–11841. 10.1073/pnas.1100532108 PMC3141934 21730153

[CIT0071] Zhang B Zhou Y 2011 Rice brittleness mutants: a way to open the ‘black box’ of monocot cell wall biosynthesis. Journal of Integrative Plant Biology 53, 136–142. 21205179 10.1111/j.1744-7909.2010.01011.x

[CIT0072] Zhong RQ Burk DH Morrison WH Ye ZH 2004 FRAGILE FIBER3, an Arabidopsis gene encoding a type II inositol polyphosphate 5-phosphatase, is required for secondary wall synthesis and actin organization in fiber cells. The Plant Cell 16, 3242–3259. 15539468 10.1105/tpc.104.027466 PMC535871

