# Supplementary material for: Molecular cloning of the tomato Hairless gene implicates actin dynamics in trichome-mediated defense and mechanical properties of stem tissue
Source: J Exp Bot. 2016 Jul 31;67(18):5313–24. doi: 10.1093/jxb/erw292 (PMC5049383; doi:10.1093/jxb/erw292)
Supplement: Supplementary Data [file supp_67_18_5313__index.html]

Molecular cloning of the tomato Hairless gene implicates actin dynamics in trichome-mediated defense and mechanical properties of stem tissue — Molecular cloning of the tomato Hairless gene implicates actin dynamics in trichome-mediated defense and mechanical properties of stem tissue — Supplementary Data 

# Molecular cloning of the tomato *Hairless* gene implicates actin dynamics in trichome-mediated defense and mechanical properties of stem tissue

## Supplementary Data

Data files

- supplementary\_figures\_S1\_S5.pdf - Supplementary Data
- supplementary\_tables\_S1\_S2.docx - Supplementary Data
- supplementary\_movie\_S1.MOV - Supplementary Data
- supplementary\_movie\_S2.MOV - Supplementary Data
- supplementary\_movie\_S3.MOV - Supplementary Data
- supplementary\_movie\_S4.MOV - Supplementary Data
