# Supplementary material for: Molecular cloning of the tomato Hairless gene implicates actin dynamics in trichome-mediated defense and mechanical properties of stem tissue
Source: J Exp Bot. 2016 Jul 31;67(18):5313–24. doi: 10.1093/jxb/erw292 (PMC5049383; doi:10.1093/jxb/erw292)
Supplement: Supplementary Data [file supp_erw292_supplementary_figures_S1_S5.pdf]

Supplementary Figures

Molecular cloning of the tomato *Hairless* gene implicates actin dynamics in trichome-mediated defense and mechanical properties of stem tissue

Jin-Ho Kang, Marcelo L Campos, Starla Zemelis-Durfee, Jameel M. Al-Haddad, A. Daniel Jones, Frank W. Telewski, Federica Brandizzi, and Gregg A. Howe

```
AtSRA1          MAVPVEEAIAALSTFSLEDEQPEVQGPAVMVSAERAATDSPIEYSDVAAYRLSLSEDTKA
SlSRA1          MAVPIEEAIAALSTFSLEDDQPEVQGPGFWVSAEGGATISPIEYSDVAAYRLSLSEDTKA
                ****:**************:*******.. **** .** *********************

AtSRA1          LNQLNTLIQEGKEMASILYTYRSCVKALPQLPESMKHSQADLYLETYQVLDLEMSRLREI
SlSRA1          INQLNTLIQEGKEMGSVLYTYRSCVKALPQLPDSMKQSQADLYLETYQVLDLEMSRLREI
                :*************.*:***************:***:***********************

AtSRA1          QRWQSSASAKLAADMQRFSRPERRINGPTVTHLWSMLKLLDVLVQLDHLKNAKASIPNDF
SlSRA1          QRWQASAASKLAADMQRFSRPERRINGPTVTHLWSMLKLLDVLIQLDHLKNAKASIPNDF
                ****:**::**********************************:****************

AtSRA1          SWYKRTFTQVSAQWQDTDTMREELDDLQIFLSTRWAILLNLHVEMFRVNNVEDILQVLIV
SlSRA1          SWYKRTFTQVSVQWQDTDSMREELDDLQIFLSTRWAILLNLHVEMFRVNNVEDILQVLIV
                ***********.******:*****************************************

AtSRA1          FIVESLELDFALLFPERYILLRVLPVLVVLATPSEKDTEALYKRVKLNRLINIFKNDPVI
SlSRA1          FIVESLELNFALLFPERHTLLRVLPVLVVLAASSEKDSESLYKRVKINRLMNIFKNDPVV
                ********:********: ************:.****:*:******:***:********:

AtSRA1          PAFPDLHLSPAAILKELSVYFQKFSSQTRLLTLPAPHELPPREALEYQRHYLIVNHIGAL
SlSRA1          PAFPDLHLSPAAILKELSTYFPKFSAQTRLLTLPAPHELPLREAQDYQRQYLIVNHIGAI
                ******************.** ***:************** *** :***:*********:

AtSRA1          RAEHDDFTIRFASSMNQLLLLKSNDGAYTEWCREVKGNMYDMVVEGFQLLSRWTARIWEQ
SlSRA1          RAEHDDFTVRFASAMSQLVLLKSIDGVDVEWVKEVKGNTYDMVVEGFQLLSRWTARVWEQ
                ********:****:*.**:**** **. .** .***** *****************:***

AtSRA1          CAWKFSRPCRDAG--ETPEASGSYSDYEKVVRFNYTAEERKALVELVGYIKSVGSMLQRC
SlSRA1          CAWKFSRPCKDPVPMESHDMPASFSDYEKVVRYNYNAEERKALVELVSYIKSIGSMMQKV
                *********.*.   *: : ..*:********:**.***********.****:***:*.

AtSRA1          DTLVADALWETIHAEVQDFVQNTLATMLRTTFRKKKDLSRILSDMRTLSADWMAN-TRPE
SlSRA1          DTSVTDALWETIHAEVQDFVQNTLATMLRTTFRKKKDLSRILSDMRTLSADWMANASKPE
                ** *:************************************************** :.**

AtSRA1          HEMPSSQHGNDESRGNFFYPRPVAPTAAQVHCLQFLIYEVVSGGNLRRPGGFFGNNGSEI
SlSRA1          TEMQSYPHSGEESRGTLFYPRPVAPTSAQVHCLQFLIYEVVSGGNMRKPGGIFGNSGSEI
                 ** *  *..:****.:*********:******************:*.***:***.****

AtSRA1          PVNDLKQLETFFYKLSFFLHILDYSASIGILTDLGFLWFREFYLESSRVIQFPIECSLPW
SlSRA1          PINDLKQLETFFYKLGFFLHVLDYTATLGTLTDLGFLWFREFYLESSRVIQFPIECSLPW
                *:*************.****:***:*::* ******************************

AtSRA1          MLIDYILEAQNSGLLESVLLPFDIYNDSAQQALVVLRQRFLYDEIEAEVDHGFDIFVSRL
SlSRA1          MLVDHVIESPIIGLLESALMSFDIYNDAAQQALVILKQRFLYDEIEAEVDNCFDIFVLKL
                **:*:::*:   *****.*:.******:******:*.*************: ***** .*

AtSRA1          SESIFTYYKSWSASELLDPSFLFALDNGEKFSIQPVRFTALFKMTKVKILGRTINLRSLI
SlSRA1          CETIFTYYKSWAASELLDPSFLFAIDIGEKFAVQPMRFVALLKTTRVKLLGRTINLRSLI
                .*:********:************:* ****::**:**.**:* *.**:***********

AtSRA1          AQRMNRIFRENLEFLFDRFESQDLCAVVELEKLIDILKHSHELLSQDLSIDPFSLMLNEM
SlSRA1          ADRMNKMFRDNLEFLFDRFESQDLCAIVELEMLLDILQLTHELLSKDLTIDSFNLMLNEM
                *:***.:**:****************:**** *:***: :*****:**:**.*.******

AtSRA1          QENISLVSFSSRLATQIWSEMQSDFLPNFILCNTTQRFVRSSKVP--PTQKPSVPSAKPS
SlSRA1          QENVSLVSYSSRLASQIWTEMQNDFLPNFILCNTTQRFVRSARVPPVPVQKPSVPYAKPN
                ***:****:*****:***:***.******************:.**  *.****** ***.

AtSRA1          FYCGTQDLNAAHQSFARLHSGFFGIPHLFSIVKLLGSRSLPWLIRALLDHISNKITTLEP
SlSRA1          FYCGTPDLNSAYQSFARLYCGFFGVPHMFSLVKLLGSRSLPWLIRALLDNISNKITTVEP
                ***** ***:*:******:.****:**:**:******************:*******:**

AtSRA1          MISGLQEALPKSIGLLSFDGGVTGCMKLIREQLN-WGTKSELKSEVLRGIKEIGSVIYTM
SlSRA1          MITGLQEALPKSIGLLPFDGGISGCMRLAKEHLSCWHSKSELKAEVLCGIKEIGSILYWM
                **:*************.****::***.* .*:*. * :*****:*** *******::* *
```

```
AtSRA1          GLLDIVLVSPVDTKRFMQTAPWLGLIPGAEGQIVNAQD-GESPLVNLLKSATSAVVSSPG
SlSRA1          GLLDIVL-REVDTRQFMQTAPWLGLIPGADGQILHSQEGGDSPMVTLFKSATTATMSNPN
                *******   ***..**************:***:::*: *:**:*.*:****:*.:*.*.

AtSRA1          CLNPAAFYTMSKQAEAADLLYKANMNGGSVLEYTLAFTSASLDKYCSKWSAPPKTGFVDI
SlSRA1          CTNPTSFHTISRQAEAADLLYKANINTGSVLEYALAFTSAALDKYCSKWSAAPKTGFIDI
                * **::*:*:*.************:* ******:******:**********.*****:**

AtSRA1          TTSKDFYRIYGGLQIGYLEEITAPQSAQHEVLGDSIAWGGCTIIYLLGQQLHFELFDFSY
SlSRA1          TTSKDFYRIFSGLQIEYLEESIQLQSNTYEMLGDSVAWGGCTIIYLLGQQLHFELFDFSH
                *********:.**** ****    **  :*:****:***********************:

AtSRA1          QVLNVSEVETVSASHTHRNPQIHQGWEGLLEGMKKARRLNNHVFSMLKARCPLEDKTACA
SlSRA1          QVLNVAEVESVAISPTQKNPNFLQGIEGLLEAMKKARRLNNHVFSMLKARCPLEDKQACA
                *****:***:*: * *:.**:: ** *****.************************ ***

AtSRA1          IKQSGAPLPRVRFENTVSAFETLPQKGTVG
SlSRA1          IKQSGAPLHRIKFENTVSAFETLPQKGA--
                ******** *:.***************:
```

Supplementary Fig. S1. Amino acid sequence comparison of Arabidopsis and tomato SRA1. Shown is a sequence alignment of Arabidopsis (*At*SRA1) and tomato (*Sl*SRA1) SRA1 proteins, which share 81% amino acid sequence identity.

(A) WT DNA

GTTGAACCTAGAATCTGTGTGCTTTGATATCTTGTGAAAATATCAGAGCATACAACTCTAAGCCTAAGAGGATTGATATAATAGCCAAGGTCTT
TATAAACCCCTATGGAAGTTCTACACAACTCATATGAAACCACTATATAACTCAACAGTTCCAGGACCTTAGCTGTTATCTGGATATATTTTAA
GCAATCACTATAGGAAGGTTGGTAAACCTAGCACATGAGATTAAGCAGAATCAAAAATCTTTGGAAAGATATCATGTGTTTGTTAAAGGGCTAC
AGTTATGCTCCTGAAAGCTGTTTTATGTAAGATCTATAAACAACAATGACAACAATGCTTTAATCCTGAACTAATAGTGGCCCTGGGCCTTCTG
CTCTTCAAATGTAAGAGGTGGCCAGCCATTTGTCAAGGCCCAGGAATCCGGTGGACATCTCAGCGGAGGGATCTAGGCATATGAATCTTTTATG
TGTTCCACTCTATTTGCCGGATTCATTCCAATGCTTAATAATGTGTCCTTTAAGATAAATTGGGGGGTTTCTAAACTTGGTACTTCTTTAGACC
TCACATATGTAAATCTGTAACCAAACTAAAAAGACTACCAACAAGTACCAGATCTTAGTCCCGGCTAATTCATATTTGCTTACTTCGACAACTA
ATGGACATTTCACATCCTCTTCAGTTCTACTCTCGGTTTGATAAGTAGAATTATCATATATTTGACATCCAACATTACTTTCTATCTTCAGGGT
ATTGAAGGTTTGCTAGAAGCTATGAAGAAAGCAAGGAGGCTAAATAATCATGTGTTTTCAATGTTAAAAGCACGCTGTCCATTGGAAGATAAAC
AGGCTTGTGCCATCAAACAAAGCGGTGCACCTTTGCATCGGATAAAATTTGAGAATACTGTGTCCGCTTTTGAAACGTTGCCACAGAAAGGTGC
CTGAGGCCTGCTTCCATTTTTCATTGCAGAAGTTTGCATGTACGAACCTTCTTATCATGTATGTAATATATGTTTATCGATGTACTCGTTGCTC
TGAGATTGAAGATGATGCTACCTGTTCAGTGATGGTATAGATATAGAGATTGTAAGATGAAGTAAAACAAGTTCATTGGAATAGATGTTGAGGC
AGCAGAATAGAACTGAACATCTAATGGAGAGGTAATAGAGTACCCATAGCATTTTTCATGAAAAGTATCTTTGAGTACATGTCAGTCTTTTCTT
TTCTTTTTCTATCAGATAAAACCTAATCATTTAGGGTAAAACAGCTTTTTGCATTGACAAATCTAAACTGGCTAAAAGCTGTAGTGTACTAACC
ACAAATGCTTTATGATACCTTTTCCATGATTGAAGGAACTATTTTTCAGGTGGAATCCTCGACTCACTTTTGTCGTTTTCTAAGCTTTTGTCAA
GAATCTACAACTATACCACTACCTGCGTCTCATTTTATATATAACATTTCGGATCTCAAAAGTCGAACAAGTTTAAA**TTTTTCATGGATTGTAT**
AGACATTTTTAATTATTAATTATTGTGATTCATGTTCTCGCTGTTTTTCAAAAGAATGAACTGGTTTGACTTGGCACGGAGTGAAAGAGTATAA
AGAAATTTTTGAATCTCGTGGTCCTAAATTAAAGTTAGATCAAATGTACAAAATTGATCTTTGATCTTGTGATCTTAAATATGCCACGTGAAAA
GTTGTACACGGATGGAGTAGTACTTTTTACGTAATTTACAAATACATAAATTTCATTTAAAAAAATTGAGGATTTTATGCGCAAATGTTCGATC
AAACATAAACCATTTGATTCTCAAAATATAAAAGATGACATATAAATTGTGATGAAATGAGTATAGTTTAATCGATTTTGTTTTACACAAATTT
TAGTGAGGAAATTGCACTAAATAATTTTCAAACCCTGATTTAAACTTAATTAGCAGCCAAAAAAGAGTATTCTGTTCTCCCCTAGTGATATACA
TACATACAAACATATAAATAACTTAATTAGATCCACACAAGCTTCTTGTATACCATTACCAATTCCAATAACATTCACTTAAATAATTTGTTGA
AGAGTAGGGCAAGATGATCAACTGTGACATTAAATGTTTTTTTTAATAAAAGAGTACAATAAATGTGAGACACATACATGAGTTGTGTTTACAG
AAAGTTATTACATTTCACCTAACTTTATATTTGGCCCTTTTTTTTTCTCTATAATAAAATGAATACTATCCTACACCACTCAAAAGAATTTAATA
CAAAATAATATCCAGCTACAACTTTAATTTGTTTGAGTCATTTACAACCATTTTTTTTTTCAAATTGGCAATTTCACTTTTAAGGAAATTGATT
TCTACACTTTGTAAGTTAGGTTTGCAATGTCCTAATGTATTGAACTTATTTTAAACACTCAAGATTAATCCTATAATCCTTCTTTATGAACTTT
AGCATAGCATTTTTATTCCTAGATAAGAAGACTTCTGTTGTGAAATTAGAAAAATACATCAACTACCAACATTATTAATGTCCACAACTACAAA
AGATGAAAAACACCTATTCACATTAAATTGTTAAAGATATTGGTCTTTGCTAAATGGGGGACCAATTTTTATACATGATATAATGATTAGCTCT
TAAGGAAACATGTCAAACAAAAAAGGGTGTGTTTAATTGGTAGGAAGAAAAATGTGTGTCACTAATATATTTATGGAAAATTAGTGATTTTTTA
ATTTATATTTTGTGTTTGGTAGGTAAGAAAAAAATATATTCCCAAAAGTATTGAATTGAATGCATGAATGGTCTGTTAACTTACAGCAACTAGT
GATCAAAGTCACTCTACTTTGCCTTTCAAAAAAGTCATCCAACTTTATCTAGTTAGCTTACAGAGTAATTTAATTGAAAATACAAATTTGTAGG
AAAACATATTTTAGATATTAAGATCAATATTTAGATTTATTTTGGACCCTGTCCCACTTAAAATGAATTGACTATAAGTTTACTGGTCAATTAT
TATACATTAACTGACCACCTAGACATTTGCAATATTTATATAATCTAGAAAAACACCATAAGGTGGATGATGGTAAGGTGTTGGGTGGAAAGAG
GGGTAAATTTACAATAATATCATGATTCGAGTCTACATCATGGACATAATCGGTATTTAAAATTATTATTGATCAGAGCAAACTAACATACTTA
AAGACTGAATAATGACTAGTGCGGAAGCTTTGAAAGATGATCGTGCAATACAATAATTTAGAGAGAAAAAAAACTCAAGAACTTTATATACATA
AAAGTCTGAAATATGTATAATATGTATATGCTTAGAAAAATAACTATGATCATAACTCAACTGGCTCGTCTATGAAGTCCCTAACATACTTTAA
AAAA**ATTATTAGGGCAAGACCCCTGGCTACCTGAACTTTACTAATAACATAAATAAATTACTAATACTCAAGATAAATTAGAGCCCTTCAAAAG**
TAAGGAGGTCTCATCATATTTGGTTAAACATGATCCTAATGATGTGTATGTCGTCCATCCTGAATATCCGAGTCTACATTATTAAAAGATGAAG
CGTAGAATGACGTAAGAAATAAAATGTAGGGTATGTAAAATAGTTGAATGAAACACTAGTTAAAATTGAAGATTCTGAGCTAAATGAATAGCTC
TGAAGATAGACTGAAATAAAGTCAGCTGAGTAGTATGAAATATGATTTTCAATTTAAAGGTTGGGAAATATT

(B) *hl* DNA

GTTGAACCTAGAATCTGTGTGCTTTGATATCTTGTGAAAATATCAGAGCATACAACTCTAAGCCTAAGAGGATTGATATAATAGCCAAGGTCTT
TATAAACCCCTATGGAAGTTCTACACAACTCATATGAAACCACTATATAACTCAACAGTTCCAGGACCTTAGCTGTTATCTGGATATATTTTAA
GCAATCACTATAGAAATGTTATTAGTAAAGTTCAGGTAGCCAGGGGTCTTGCCCTAATAATTTTTCATGGATTGTATAGACATTTTTAATTAT
TAATTATTGTGATTCATGTTCTCGCTGTTTTTCGATGTACTCGTTGCTCTGAGATTGAAGATGATGCTACCTGTTCAGTGATGGTATAGAAATA
AATTACTAATACTCAAGATAAATTAGAGCCCTTCAAAAGTAAGGAGGTCTCATCATATTTGGTTAAACATGATCCTAATGATGTGTATGTCGTC
CATCCTGAATATCCGAGTCTACATTATTAAAAGATGAAGCGTAGAATGACGTAAGAAATAAAATGTAGGGTATGTAAAATAGTTGAATGAAACA
CTAGTTAAAATTGAAGATTCTGAGCTAAATGAATAGCTCTGAAGATAGACTGAAATAAAGTCAGCTGAGTAGTATGAAATATGATTTTCAATTT
AAAGGTTGGGAAATATT

Supplementary Fig. S2. Genomic sequence of the affected region in WT and *hl* plants.

(A) Sequence of WT *SRA1* genomic DNA. The red highlighted sequence denotes the last exon of the gene. Sequences that are present or absent (3187 bp) in *hl* are indicated by the black and gray letters, respectively. Sequences that are present but rearranged in *hl* are underlined in red (56 bp), dark blue (66 bp), and green (46 bp). (B) Sequence of *SRA1* genomic DNA in the *hl* mutant. WT sequences that are retained in *hl* are underlined in red (56 bp), dark blue (66 bp), and green (46 bp), and correspond to the underlined sequences in panel A. The orientation of sequences underlined in green is reversed in WT. In addition, two nucleotides (highlighted with red) appear to be inserted in *hl*.

```
WT          MAVPIEEAIAALSTFSLEDDQPEVQGPGFWVSAEGGATISPIEYSDVAAYRLSLSEDTKA
hl          MAVPIEEAIAALSTFSLEDDQPEVQGPGFWVSAEGGATISPIEYSDVAAYRLSLSEDTKA
            ************************************************************

WT          INQLNTLIQEGKEMGSVLYTYRSCVKALPQLPDSMKQSQADLYLETYQVLDLEMSRLREI
hl          INQLNTLIQEGKEMGSVLYTYRSCVKALPQLPDSMKQSQADLYLETYQVLDLEMSRLREI
            ************************************************************

WT          QRWQASAASKLAADMQRFSRPERRINGPTVTHLWSMLKLLDVLIQLDHLKNAKASIPNDF
hl          QRWQASAASKLAADMQRFSRPERRINGPTVTHLWSMLKLLDVLIQLDHLKNAKASIPNDF
            ************************************************************

WT          SWYKRTFTQVSVQWQDTDSMREELDDLQIFLSTRWAILLNLHVEMFRVNNVEDILQVLIV
hl          SWYKRTFTQVSVQWQDTDSMREELDDLQIFLSTRWAILLNLHVEMFRVNNVEDILQVLIV
            ************************************************************

WT          FIVESLELNFALLFPERHTLLRVLPVLVVLAASSEKDSESLYKRVKINRLMNIFKNDPVV
hl          FIVESLELNFALLFPERHTLLRVLPVLVVLAASSEKDSESLYKRVKINRLMNIFKNDPVV
            ************************************************************

WT          PAFPDLHLSPAAILKELSTYFPKFSAQTRLLTLPAPHELPLREAQDYQRQYLIVNHIGAI
hl          PAFPDLHLSPAAILKELSTYFPKFSAQTRLLTLPAPHELPLREAQDYQRQYLIVNHIGAI
            ************************************************************

WT          RAEHDDFTVRFASAMSQLVLLKSIDGVDVEWVKEVKGNTYDMVVEGFQLLSRWTARVWEQ
hl          RAEHDDFTVRFASAMSQLVLLKSIDGVDVEWVKEVKGNTYDMVVEGFQLLSRWTARVWEQ
            ************************************************************

WT          CAWKFSRPCKDPVPMESHDMPASFSDYEKVVRYNYNAEERKALVELVSYIKSIGSMMQKV
hl          CAWKFSRPCKDPVPMESHDMPASFSDYEKVVRYNYNAEERKALVELVSYIKSIGSMMQKV
            ************************************************************

WT          DTSVTDALWETIHAEVQDFVQNTLATMLRTTFRKKKDLSRILSDMRTLSADWMANASKPE
hl          DTSVTDALWETIHAEVQDFVQNTLATMLRTTFRKKKDLSRILSDMRTLSADWMANASKPE
            ************************************************************

WT          TEMQSYPHSGEESRGTLFYPRPVAPTSAQVHCLQFLIYEVVSGGNMRKPGGIFGNSGSEI
hl          TEMQSYPHSGEESRGTLFYPRPVAPTSAQVHCLQFLIYEVVSGGNMRKPGGIFGNSGSEI
            ************************************************************

WT          PINDLKQLETFFYKLGFFLHVLDYTATLGTLTDLGFLWFREFYLESSRVIQFPIECSLPW
hl          PINDLKQLETFFYKLGFFLHVLDYTATLGTLTDLGFLWFREFYLESSRVIQFPIECSLPW
            ************************************************************

WT          MLVDHVIESPIIGLLESALMSFDIYNDAAQQALVILKQRFLYDEIEAEVDNCFDIFVLKL
hl          MLVDHVIESPIIGLLESALMSFDIYNDAAQQALVILKQRFLYDEIEAEVDNCFDIFVLKL
            ************************************************************

WT          CETIFTYYKSWAASELLDPSFLFAIDIGEKFAVQPMRFVALLKTTRVKLLGRTINLRSLI
hl          CETIFTYYKSWAASELLDPSFLFAIDIGEKFAVQPMRFVALLKTTRVKLLGRTINLRSLI
            ************************************************************

WT          ADRMNKMFRDNLEFLFDRFESQDLCAIVELEMLLDILQLTHELLSKDLTIDSFNLMLNEM
hl          ADRMNKMFRDNLEFLFDRFESQDLCAIVELEMLLDILQLTHELLSKDLTIDSFNLMLNEM
            ************************************************************

WT          QENVSLVSYSSRLASQIWTEMQNDFLPNFILCNTTQRFVRSARVPPVPVQKPSVPYAKPN
hl          QENVSLVSYSSRLASQIWTEMQNDFLPNFILCNTTQRFVRSARVPPVPVQKPSVPYAKPN
            ************************************************************

WT          FYCGTPDLNSAYQSFARLYCGFFGVPHMFSLVKLLGSRSLPWLIRALLDNISNKITTVEP
hl          FYCGTPDLNSAYQSFARLYCGFFGVPHMFSLVKLLGSRSLPWLIRALLDNISNKITTVEP
            ************************************************************
```

```
WT              MITGLQEALPKSIGLLPFDGGISGCMRLAKEHLSCWHSKSELKAEVLCGIKEIGSILYWM
hl              MITGLQEALPKSIGLLPFDGGISGCMRLAKEHLSCWHSKSELKAEVLCGIKEIGSILYWM
                ************************************************************

WT              GLLDIVLREVDTRQFMQTAPWLGLIPGADGQILHSQEGGDSPMVTLFKSATTATMSNPNC
hl              GLLDIVLREVDTRQFMQTAPWLGLIPGADGQILHSQEGGDSPMVTLFKSATTATMSNPNC
                ************************************************************

WT              TNPTSFHTISRQAEAADLLYKANINTGSVLEYALAFTSAALDKYCSKWSAAPKTGFIDIT
hl              TNPTSFHTISRQAEAADLLYKANINTGSVLEYALAFTSAALDKYCSKWSAAPKTGFIDIT
                ************************************************************

WT              TSKDFYRIFSGLQIEYLEESIQLQSNTYEMLGDSVAWGGCTIIYLLGQQLHFELFDFSHQ
hl              TSKDFYRIFSGLQIEYLEESIQLQSNTYEMLGDSVAWGGCTIIYLLGQQLHFELFDFSHQ
                ************************************************************

WT              VLNVAEVESVAISPTQKNPNFLQGIEGLLEAMKKARRLNNHVFSMLKARCPLEDKQACAI
hl              VLNVAEVESVAISPTQKNPNFLQV------------------------------------
                ***********************

WT              KQSGAPLHRIKFENTVSAFETLPQKGA
hl              ---------------------------
```

Supplementary Fig. S3. Comparison of the predicted amino acid sequence of SRA1 in WT and *hl* plants. The *hl* mutant form of SRA1 is predicted to lack 63 amino acids at the C-terminus of the protein.

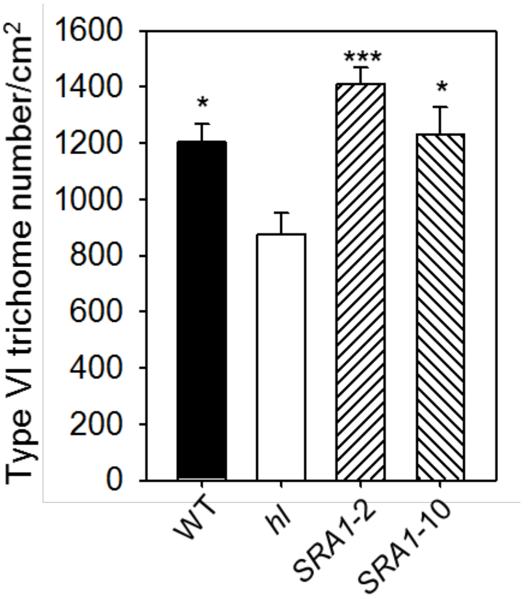

Supplementary Fig. S4. Density of type VI trichomes on leaves from WT, *hl*, and *SRA1*-complemented transgenic lines.

Light microscopy was used to determine the density of type VI trichomes on leaves of 4-week-old WT, *hl*, and complemented transgenic lines (*SRA1*-2 and *SRA1*-10). Each data point represents the mean ± SE of five independent plants per line. Asterisks represent significant differences between *hl* and other genotypes (unpaired *t* test: * $P < 0.05$; *** < 0.001).

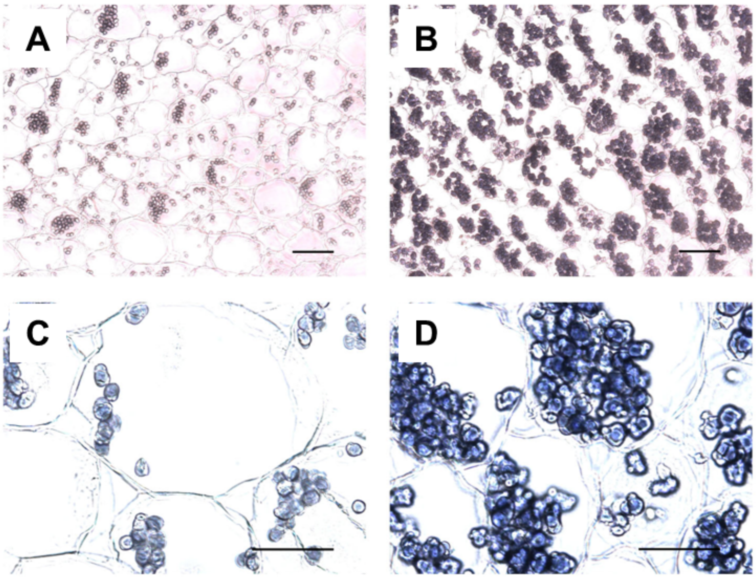

Supplementary Fig. S5. Iodine staining of pith cells from WT and *hl* stems.

WT (A) and *hl* (B) pith cells at low magnification (scale bar: 100 µm). WT (C) and *hl* (D) pith cells at high magnification (scale bar: 50 µm). Iodine-stained granules show characteristic purple-black color.
