# Supplementary material for: Molecular cloning of the tomato Hairless gene implicates actin dynamics in trichome-mediated defense and mechanical properties of stem tissue
Source: J Exp Bot. 2016 Jul 31;67(18):5313–24. doi: 10.1093/jxb/erw292 (PMC5049383; doi:10.1093/jxb/erw292)
Supplement: Supplementary Data [file supp_erw292_supplementary_tables_S1_S2.docx]

Kang et al., (2016) Molecular cloning of the tomato *Hairless* gene implicates actin dynamics in trichome-

mediated defense and mechanical properties of stem tissue

| **Supplementary Table S1.** Description of PCR-based mapping markers. | | |  |  |  |
| --- | --- | --- | --- | --- | --- |
| **Marker name** | **Forward primer sequence**  **(5’🡪3’)** | **Reverse primer sequence**  **(5’🡪3’)** | **PCR product size (bp)** | **Mapping enzyme** | **Digest size (bp)** |
|  |  |  | ***S. lycopersicum***  ***/S. pennellii*** |  | ***S. lycopersicum***  ***/S. pennellii*** |
| U565097 | ATTTTGAAAAGACAGTGG | TCACCACCACCAAAATTAC | 121/120 | *Hpy188*III | 121/73, 47 |
| U601668 | AACCCGAACGAAAGTTGAG | AGTTTGATGGAGCAGTAG | 131/130 | *EcoR*I | 73, 58/130 |
| U576269 | TGGTGGTTTCTTTAATGGTG | ACAGAAAACATCCTCAGTG | 164/163 | *Hinf*I | 164/97, 66 |
| T0675 | TTGTGAGGTTTACCTTCTGC | TAAATCTAAATAATAGCACTT | 248/248 | *Dra*I | 248/178, 70 |
| C2_At1g44790 | TCGGTTTTATCAAAGGCTATCGTC | TGTTACTGTTCTACCTGGGAATTCTGG | 300/250 |  |  |

| **Supplementary Table S2.** Description of PCR primers used in this study. | |  |  |
| --- | --- | --- | --- |
| **Primer Name** | **Primer sequence (5’🡪3’)** | **Description** |  |
| PIR-cDNA | F: TCAA**ATG**GCGGTTCCGATTGAAGAAG  R: AGCAGGCC**TCA**GGCACCTTTCTGTGGC | Amplifies full-length *SRA1* cDNA | |
| eIF4A | F: GAAAGACGGCAACTTTCTGC  R: GAGCCTCTGGTGGCATAGTC | Control for RT-PCR | |
| gPIR1 | F: CTAGTAAAAGTGAAAGCACG  R: CCACCACATATTGTAATCATTTTC | Amplifies genomic region containing *SRA1* exons 1 to 4 | |
| gPIR2 | F: CCATGTTTTTGCCACTGTCC  R: CAAGGATGGAAAAGTTGAGC | Amplifies genomic region containing *SRA1* exons 5 to 7 | |
| gPIR3 | F: GTCCACTAGACTAGTTAGGG  R: GATAGCAGGTCAGGTCTAGC | Amplifies genomic region containing *SRA1* exons 8 and 9 | |
| gPIR4 | F: GAGTATAGCACAAATGATGG  R: AGATAATAAGGACATTTTCC | Amplifies genomic region containing *SRA1* exon 10 | |
| gPIR5 | F: ACTGTACTTTTGAGACACTGG  R: TATGTGTGTGACTTGTGAGAG | Amplifies genomic region containing *SRA1* exons 11 and 12 | |
| gPIR6 | F: TAAGCGGAGAAGGGTAGAGG  R: GTATGAATATTGTACATATGAG | Amplifies genomic region containing *SRA1* exons 13 and 14 | |
| gPIR7 | F: TGATTATGATTCTTCTAGTC  R: GATCAAGAATGTTTGTCTGCC | Amplifies genomic region containing *SRA1* exons 15 and 16 | |
| gPIR8 | F: CAATTCCATGTACTCCCTGC  R: ATTAATATGCAAATGTAACG | Amplifies genomic region containing *SRA1* exons 17 and 18 | |
| gPIR9 | F: TAGTCGTCTCAATAGAAAGC  R: CTGGTAAAACAGATAAGTTC | Amplifies genomic region containing *SRA1* exons 19 and 20 | |
| gPIR10 | F: TGTGAGAATCTTGGTTTACC  R: CATATAGGAAATCAGTGAAG | Amplifies genomic region containing *SRA1* exons 21 and 22 | |
| gPIR11 | F: GATAAGAAAGTTGTCAATGC  R: TTGCATTAGTAAGATTTAGC | Amplifies genomic region containing *SRA1* exon 23 | |
| gPIR12 | F: CCCTTGTTTATTGTTCTTGC  R: TTTAGAGTGAGAAGCTCAGG | Amplifies genomic region containing *SRA1* exons 24 to 26 | |
| gPIR13 | F: ATTGAAGTAAATTAACTTGG  R: CATGTCTGAAATAATATTGACG | Amplifies genomic region containing *SRA1* exon 27 | |
| gPIR14 | F: TCAGCTTTCTTCTTGTATCG  R: GATTCTTACAGATGCAGTTC | Amplifies genomic region containing *SRA1* exons 28 and 29 | |
| gPIR15 | F: ATGTGTTCCACTCTATTTGC  R: ACGACAAAAGTGAGTCGAGG | Amplifies genomic region containing *SRA1* exon 30 | |
| 3'RACERT | R: GCTCGCGAGCGCGTTTAAACGCGCACGCGTTTTTTTTTTTTTTTTTT | Used for 3' RACE reverse transcriptase reaction | |
| 3'RACE1 | F: AGATTACTACCGTTGAACCG  R: GCTCGCGAGCGCGTTTAAAC | Used for 3' RACE (gene specific): from 25th exon to 3' end | |
| 3'RACENESTED | F: CGTTGAACCGATGATTACTG  R: GCGTTTAAACGCGCACGCGT | Used for 3' RACE nested PCR (gene specific): from 25th exon to 3' end | |
| PIR-SX | F: CGACTAGTATGGCGGTTCCGATTGAA  R: TTCTCGAGTCAGGCACCTTTCTGTGG | Used to clone *SRA1* into binary vector pBI-TS. Forward and reverse primers contain *Spe*I and *Xho*I site, respectively. | |
| 35S-PIR | F: TGCCATCATTGCGATAAAGG  R: CACGGAACATCTCAACATGC | Used to amplify the region spanning the CaMV promoter and *SRA1* cDNA. |  |
| Start and stop codons are in bold. Restriction enzyme sites are underlined. | | |  |
